# Maximizing biomarker discovery by minimizing gene signatures

**DOI:** 10.1186/1471-2164-12-S5-S6

**Published:** 2011-12-23

**Authors:** Chang Chang, Junwei Wang, Chen Zhao, Jennifer Fostel, Weida Tong, Pierre R Bushel, Youping Deng, Lajos Pusztai, W Fraser Symmans, Tieliu Shi

**Affiliations:** 1 The Center for Bioinformatics and the Institute of Biomedical Sciences, School of Life Science, East China Normal University, 500 Dongchuan Road, Shanghai 200241, China; 2 SRA Global Health Sector/NIEHS, Research Triangle Park, NC, 27709, USA; 3 National Center for Toxicological Research, US Food and Drug Administration, 3900 NCTR Road, Jefferson, AK 72079, USA; 4 Biostatistics Branch, National Institute of Environmental Health Sciences, P.O. Box 12233, Research Triangle Park, NC 27709, USA; 5 Rush University Cancer Center, Department of Internal Medicine, Rush University Medical Center, Chicago, IL 60612, USA; 6 Department of Breast Medical Oncology and Department of Pathology, The University of Texas M. D. Anderson Cancer Center, PO Box 301439, Houston, TX 77230, USA

## Abstract

**Background:**

The use of gene signatures can potentially be of considerable value in the field of clinical diagnosis. However, gene signatures defined with different methods can be quite various even when applied the same disease and the same endpoint. Previous studies have shown that the correct selection of subsets of genes from microarray data is key for the accurate classification of disease phenotypes, and a number of methods have been proposed for the purpose. However, these methods refine the subsets by only considering each single feature, and they do not confirm the association between the genes identified in each gene signature and the phenotype of the disease. We proposed an innovative new method termed Minimize Feature's Size (MFS) based on multiple level similarity analyses and association between the genes and disease for breast cancer endpoints by comparing classifier models generated from the second phase of MicroArray Quality Control (MAQC-II), trying to develop effective meta-analysis strategies to transform the MAQC-II signatures into a robust and reliable set of biomarker for clinical applications.

**Results:**

We analyzed the similarity of the multiple gene signatures in an endpoint and between the two endpoints of breast cancer at probe and gene levels, the results indicate that disease-related genes can be preferably selected as the components of gene signature, and that the gene signatures for the two endpoints could be interchangeable. The minimized signatures were built at probe level by using MFS for each endpoint. By applying the approach, we generated a much smaller set of gene signature with the similar predictive power compared with those gene signatures from MAQC-II.

**Conclusions:**

Our results indicate that gene signatures of both large and small sizes could perform equally well in clinical applications. Besides, consistency and biological significances can be detected among different gene signatures, reflecting the studying endpoints. New classifiers built with MFS exhibit improved performance with both internal and external validation, suggesting that MFS method generally reduces redundancies for features within gene signatures and improves the performance of the model. Consequently, our strategy will be beneficial for the microarray-based clinical applications.

## Background

A condition's gene signature is defined as the group of genes in a given cell type whose combined expression pattern is uniquely characteristic of that condition [1]. The use of gene signatures can potentially be of considerable value in the field of clinical diagnosis. However, gene signatures defined by different investigators using different methods can be quite various even when applied on the same disease and the same endpoint. Therefore, it brings noise to the microarray-based clinical applications. For example, in the second phase of the MicroArray Quality Control (MAQC-II) project [2], a total of 19 780 gene signatures were defined by over 30 data analysis teams (DATs) for 13 endpoints. Interestingly, the genes identified in each gene signature were different for each endpoint, with some of the signatures failing to share any gene in common. However, despite the variability of these gene signatures, they still have relatively good predictable power. Then an important question is raised that why so many gene signatures can be selected for the same disease with similar predictive performance. Whether there is any signature that contains the smallest number of genes and has good performance at the same time?

Previous studies have shown that the correct selection of subsets of genes from microarray data is key for the accurate classification of disease phenotypes [3], as this procedure not only removes features that do not provide significant incremental information, but also enables more rapid and efficient analysis [4]. To this end, a number of studies have been proposed [3,5-9]. One of them is the so-called minimum redundancy-maximum relevance (MRMR). This method employs features that are maximally dissimilar to each other in terms of Euclidean distances or pair-wise correlations [3]. Based on MRMR method, Incremental Feature Selection (IFS) has been employed to determine how many features in the list MRMR generated should be selected [5]. An alternative strategy, called joint core genes, makes use of two independent lung cancer microarray datasets [6] to increase robustness of prediction. Sparse linear programming (SPLP) [10] represents a third approach which has been applied to a large microarray dataset derived analyzing from liver gene expression of compound-treated rats. In this third approach, a necessary gene set (NGS) is constructed through a stripping procedure, after which no valid signature can be derived from its complement (i.e. all genes present on the array minus the NGS) [7]. Support Vector Machine methods based on Recursive Feature Elimination (SVMRFE) refine the optimum feature set by using SVM-train to compute the ranking criteria, which eliminate the feature with smallest ranking criterion [8,9]. Like SVMRFE, recursive feature addition (RFA) employs supervised learning, and combines it with statistical similarity measures [9]. However, these methods refine the subsets by only considering each single feature. Furthermore, none of them have confirmed the fundamental association between the genes identified in each gene signature and the phenotype of the disease, which is considered to be important in clinical applications.

MAQC-II is a collaborative research project that includes individuals from multiple data analysis teams (DATs) to generate gene expression signatures for three clinical datasets and three toxicogenomics datasets [2], and offers a valuable chance for studying the relationship between gene signatures and their genes. Each DAT has the freedom to choose their own method for signature development, which has lead to many different signatures with similar predictive power for the same endpoint. The diversity of the gene signatures being largely attributed to the different modeling processes [11-13] that has been applied to the data. Consequently, these MAQC-II gene expression signatures offer a unique opportunity to develop novel meta-analysis strategies that yield a minimum set of features with maximum predictive power.

In this study we have sought to develop effective meta-analysis strategies to transform the MAQC-II signatures into a robust and reliable set of biomarker for clinical applications.

**Figure 1:**
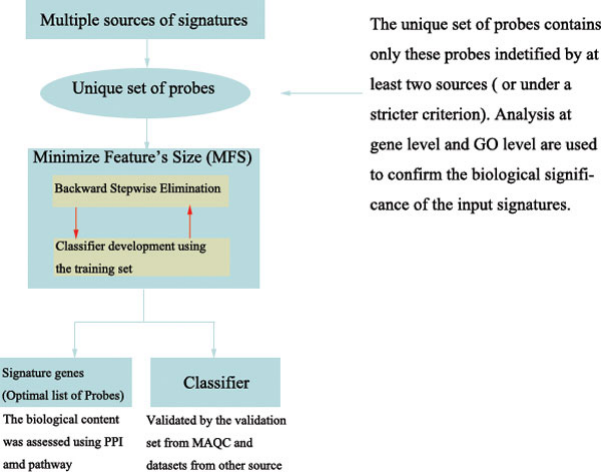
**Analysis workflow**. This figure illuminates the general outline of the whole process.

The analyses workflow is outlined in Figure 1. Specifically, we first conducted signature similarity analyses at probe level, which can select probes that are consistently considered by multiple signatures, results in a subset of features (probes) that were further analyzed using a method designated Minimize Feature's Size (MFS). MFS is an iterative backward stepwise elimination method with the purpose of minimizing the gene set (i.e., removing uninformative genes while retaining these essential to the endpoint studied) and thus generating a signature of smaller size and improving predictive power based on the resultant feature set. On the other hand, analyses at gene level and gene ontology (GO) level were proposed to confirm biological significance.

The analyses are focused on two endpoints of the MAQC-II breast cancer datasets, i.e., pathologic complete response (denoted as endpoint D hereafter) and estrogen receptor status (denoted as endpoint E hereafter). The datasets contain a training dataset and a validation dataset [14]. The minimized signatures were built on the training dataset and be validated on the validation dataset. Correspondingly, the swap models are signatures built on validation dataset and be validated on the training dataset. It is worthwhile to mention that the training and validation datasets are profiled separately in different timeframes, thus providing a real-world clinical application scenario.

## Results

### Similarity analyses at probe level

Thirty-two gene signatures for endpoint D and 22 for endpoint E were used for the similarity analyses. Overlap matrices at probe level for each of two signatures were constructed (Additional file 1 and 2). For endpoint D, 345 probes appeared at least twice among a total set of 1 747 (19.75%), whereas for endpoint E, 644 probes appeared among 1 760 (36.59%) (Table 1). The gene *ESR1 *or *estrogen receptor 1 *(Probe 205225_at) appears 21 and 19 times in endpoints D and E, respectively, ranking top 1 in both groups (Additional file 3). However, the comparison among all the involved models for each endpoint revealed that not all DATs chose the most differentially expressed probes, indicating that the selection criteria were not consistent (Additional file 4). Further analyses about probesets for both endpoints are available in Additional File 5.

**Table 1:** Overlap at the levels of probes and genes

				Probe				Gene			
Models	Endpoint	Model Number	Mean (Variance)	Total	Overlapped	Rate(%)	Endpoint Overlap	Total	Overlapped	Rate(%)	Endpoint Overlap
Normal	D	28	97.04 (91310.26)	1747	345	19.75	785	1350	402	29.78	619
	E	22	143.5 (95148.74)	1760	644	36.59		1309	589	45.00	
Swap	D	20	54.10 (3964.62)	609	207	33.99	317	465	174	37.42	252
	E	20	106.05 (32074.79)	1047	443	42.31		793	416	52.46	

This table shows prime analyses at levels of probes and genes for normal models and swap models. *Total *refers to the total number of irredundant probes and genes, with means and variances of models for each endpoint be listed in *Mean (Variance)*. *Rate *refers to the proportion of overlaps in unique sets of probes and genes. The numbers of overlaps between unique sets of probes and genes of both endpoints are calculated, which are displaced in *Endpoint Overlap*. Note that two probes for NIEHS_BR_E_5 were removed as they do not appear in the Affymetrix U133A platform. We compared lists of probes for each group of data to get overlaps at probe level. If two probes overlapped at probe level, they must also overlap at gene level; if two probes are not same but they share the same gene, they can also overlapped at gene level. This table suggested that the overlap rates of endpoint E are always greater than those of endpoint D. For normal models, the number of features for both endpoints have no significant difference (F = 0.9507, T-test = 0.5748) and there is either no significant difference for that of swap models (F = 0.1236, T-test = 1.2238, degree of freedom = 23.6263). Furthermore, overlap rates at gene level are greater than those at probe level, suggested that the number of non-identical probes which share the same genes is large.

### Model development based on the optimal set of features and the validation

The minimized signatures were built by MFS based on the result of similarity analysis at probe level. The parameters and performances of our new models, BR_D_Model and BR_E_Model, are listed in Table 2. For each endpoint, Matthew's correlation coefficient (MCC) of internal validation was improved using the MFS method, and values of validation dataset also performed well. The intervals of external validation values for all input classifiers were [-0.2482, 0.3863] (mean = 0.2702, variance = 0.0213) for endpoints D and [0.499, 0.792] (mean = 0.7105, variance = 0.0062) for endpoint E, respectively; the maximum value in both endpoints was slightly smaller than those of BR_D_Model (0.395) and BR_E_Model (0.819). Further external validations on endpoint E with EV1 dataset [15-17] and EV2 dataset [18] also verified our model (Additional file 6). Swap predictions (reverse) were also carried out between validation dataset and training dataset based on swap models submitted by DATs. However, the results of two new models from swap validations (Swap_BR_D_Model and Swap_BR_E_Model) were not as good as the independent validations of the best model (Table 2).

**Table 2:** Model parameters and performances

UniqueModelID	BR_D_Model	Swap_BR_D_Model	BR_E_Model	SwapBR_E_Model
Endpoint	D	D	E	E
Dataset	training dataset	validation dataset	training dataset	validation dataset
Samples	130	100	130	100
Features	32	33	55	10
Normalization	MAS5	MAS5	MAS5	MAS5
Batch Effect Removal Method	AGC	AGC	none	None
Feature Selection Method	MCC-robustness	MCC-robustness	MCC-robustness	MCC-robustness
Classification Method	SVM	SVM	SVM	SVM
Internal Validation	5F-CV	5F-CV	5F-CV	5F-CV
Validation Iterations	10	10	10	10
MFS Fitting Index	index1	index1	MCC	MCC
MFS Optimized Method	SVM	SVM	SVM	SVM
MFS Best Fitting Model	yes	yes	yes	yes
CV_MCC	0.707	0.689	0.904	0.942
CV_ACC	0.892	0.827	0.955	0.972
CV_SEN	0.915	0.673	0.947	0.955
CV_SPE	0.815	0.981	0.959	0.983
MCC_Std Dev	0.030	0.082	0.029	0.021
ACC_Std Dev	0.011	0.048	0.014	0.010
SEN_Std Dev	0.011	0.091	0.017	0.024
SPE_Std Dev	0.026	0.013	0.013	0.000
Val_MCC	0.395	0.368	0.819	0.661
Val_ACC	0.850	0.792	0.910	0.838
Val_SEN	0.907	0.714	0.841	0.914
Val_SPE	0.500	0.802	0.964	0.811

ACC is short for Accuracy, SEN for Sensitivity, SPE for specificity and StdDev for standard deviation. The CV rows refer to internal validation and the Val rows refer to validation of the training dataset against the validation dataset. We balanced the training dataset for Swap_BR_D_Model, as the P/N ratio is too small (0.18), as MCC is very sensitive and its value might change a lot even for a small predictive error in P/N ratio (positive/negative ratio) unbalanced datasets. Features of BR_D_Model and BR_E_Model are available at Additional file 17.

**Figure 2:**
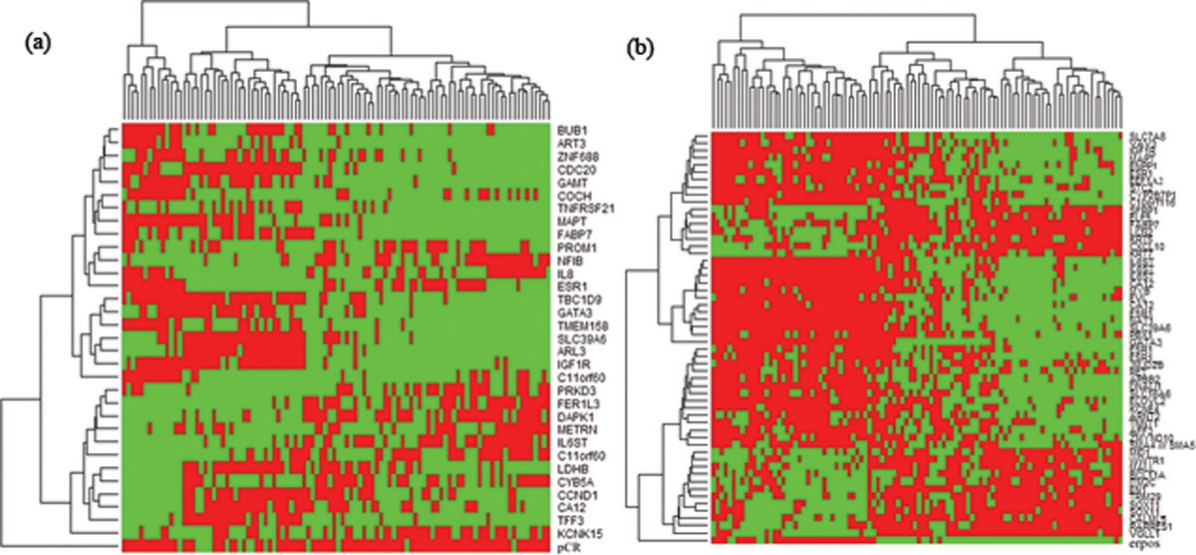
**Heatmaps for gene signatures on validation dataset**. (a) Heatmap for BR_D_Model; (b) Heatmap for BR_E_Model. Each column represents a sample in the dataset, and each row represents a gene in the gene signature. Note that the end row is endpoint status.

**Figure 3:**
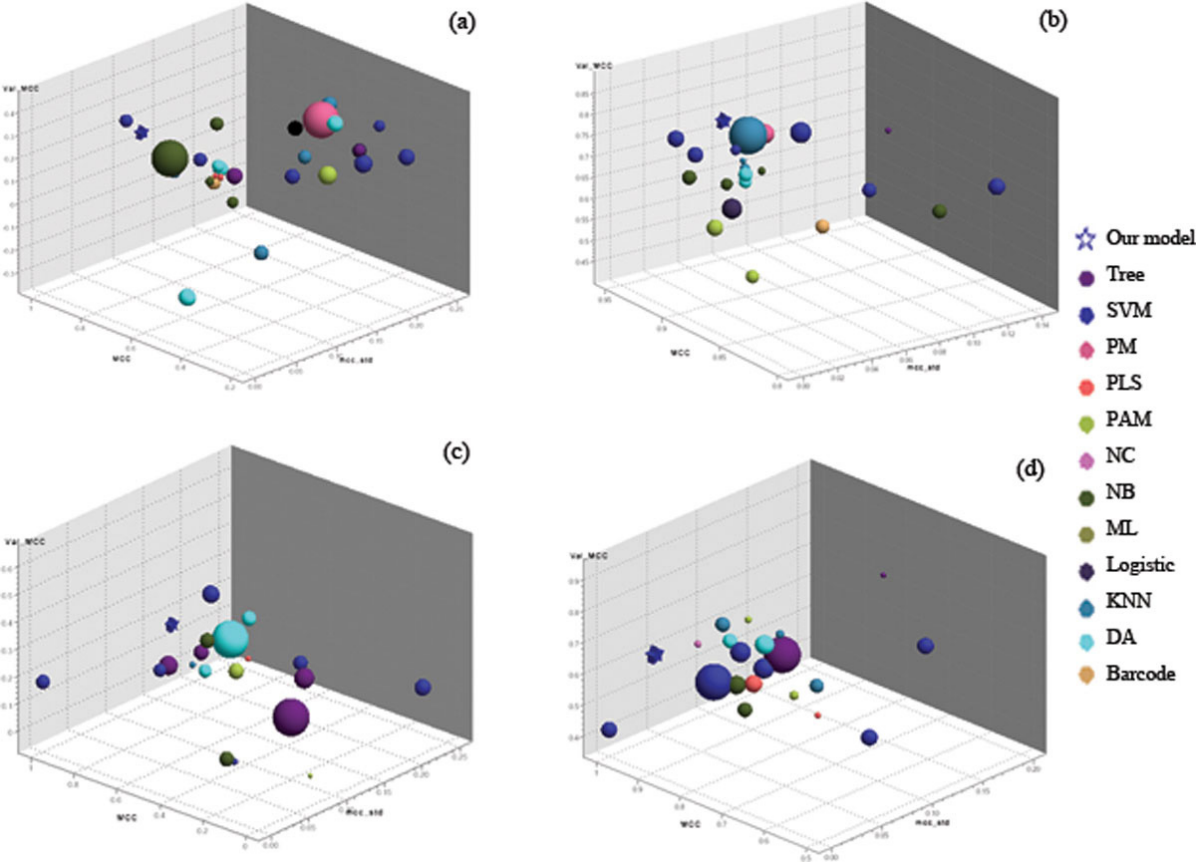
**Performances of original and swap models based on classification algorithm level similarity analysis**. a) Endpoint D original models; b) Endpoint E original models; c) Endpoint D swap models; and d) Endpoint E swap models. Coordinate axes are MCC (internal validation), Val_MCC (external validation) and MCC_Std (internal validation standard deviation). Each classification algorithm is represented by a different color. The radius of each sphere is related to the number of model features, within a range of 50-1 000. The blue stars are our own models, while spheres are models from which our models were developed.

To systematically evaluate the performances of these original models and swap models, heatmaps for these signatures on training dataset and validation dataset were created (Figure 2 andAdditional file 7). Furthermore, a three-dimensional graph was plotted, based on values of their MCC, Val_MCC (Validation MCC) and Std_Dev (standard deviation). A model that is located at upper left corner in this graph tends to have better MMC and Val_MCC with small standard deviation, and better performance. Our models for two endpoints are located at upper left corner and on the top of all other models displayed in the graphs (For BR_D_Model, MCC = 0.707, Val_MCC = 0.395, Std_Dev = 0.030; For BR_E_Model, MCC = 0.904, Val_MCC = 0.819, Std_Dev = 0.029) (Table 2 and Figure 3).

### Biological significance among signatures

To identify the biological significance among signatures, we first performed the similarity analyses at gene level and GO level. The number of genes shared by different signatures for each endpoint was quite large, and a gene can be selected more than once by a signature since multiple probes on the microarray can map to a single gene. For endpoints D and E, 29.8% and 45.0% of the total genes appeared in common, among which *CA12 *(*carbonic anhydrase XII*) related probes appeared 67 and 61 times, respectively (Table 3 and additional file 8). Then we carried out GO enrichment analysis using the GoMiner algorithm [19], and GO terms were sorted according to the number of genes associated within the 5th level (Additional file 9). The enrichment results of biological process for both endpoints are regulation of cellular metabolic process, and those of cellular component for both are intracellular part. The results of molecular function are sequence specific DNA binding and actin binding for endpoints D and E, respectively.

**Table 3:** Biological associations between genes and breast cancer

Gene	General description	Support	Endpoint D		Endpoint E	
			Overlap	Position	Overlap	Position
*CA12*	Highly correlated with Erα	+	67	-5	61	4
*MAPT*	prognostic values in ER(+) Primary breast cancers in 3 patient cohorts	+	43	-7	22	26
*ESR1*	Involved in pathological processes of breast cancer	+	33	-2	53	1
*GATA3*	A breast cancer marker	+	25	-14	40	6
*BTG3*	Protein coding		22	75		-142
*NFIB*	Differentially expressed reported [39]		22	84	20	-61
*LDHB*	Protein coding		21	27		-239
*RARRES1*	Downregulated reported [40]		20	2	19	-2
*CCND1*	Aberrantly regulated and could contribute to therapeutic failure in the context of ER-positive breast cancer	+	18	-56		145
*ELF5*	Downregulated reported [41]		16	7		-16
*IL6ST*	Protein coding			-60	33	60
*SLC39A6*	Coregulated with estrogen receptor in some breast cancers	+		-64	25	41
*TBC1D9*	Protein coding			-25	25	19
*ABAT*	Protein coding			-34	16	29

Genes marked '+' indicates experimental support for a role in breast cancer in *Support*. Unless otherwise stated, descriptions were collected from Entrez Gene [25]. Numbers in the *Overlap *reflect number of gene overlap, for the top 10 ranked genes of two endpoints. Values for *Position *reflect these genes' rank in the descending sorted list of fold-change values, signs of which represent gene positions in the two ends of the list. Almost all top 10 genes for each endpoint rank in the top 200 up-regulated or down-regulated genes. Furthermore, among the genes common to these two endpoints, the change in expression level was not always correlated. In other words, genes up-regulated in endpoint D could be down-regulated in endpoint E.

To further validate the biological significance, we analyzed the similarity between two endpoints at probe and gene levels. It was found that 27.61% of probes (214) were common to both endpoints; 16.90% of probes (131) were unique for endpoint D, while 55.48% (430) were unique for endpoint E. Comparisons at the gene level gave similar results. About 31% of genes (236) were common to both groups, while 21.99% (166) and 46.75% (353) of genes were unique to endpoints D and E, respectively. Interestingly, these two endpoints for breast cancer share a large number of overlapping probes and genes.

Noticeably, the observation that the two endpoints for the same disease share a lot of genes raised the possibility that the gene signatures for both endpoints could be interchangeable. We tested this hypothesis by cross-prediction with two minimized signatures, i.e. signatures for endpoint D were used for prediction tasks of endpoint E and *vice versa*. Interestingly, subsequent analysis results confirmed our hypothesis, although these two endpoints represent two different aspects of breast cancer. With the model BR_D_Model to predict the endpoint E, we obtained a MCC of 0.839 for internal validation, and 0.719, 0.715 and 0.727 for validation datasets, EV1 dataset and EV2 dataset, respectively. With BR_E_Model to endpoint D, the internal validation result is 0.142, and validation result for validation dataset is 0.349. Moreover, clinical data show that associations between these two endpoints were significant [20], and this association was confirmed by Kendall's rank correlation test (z = -5.9038, P = 3.551 × 10^-9^) with MAQC-II breast cancer datasets.

Furthermore, protein-protein interaction (PPI) topology property changed marginally between probes of the minimized signatures and their input, which is also a support to biological significance. All-pairs shortest path matrices [21] were constructed for each pair of probes in the minimized gene signatures based on a unique PPI network data archived at UniHI for Affymetrix probes [22]. That is, from the node of the first probe in a pair, we searched for the second one in the network using the Breadth First Search (BFS) algorithm [21]. For each pair within the matrix, three outcomes were potential: (1) one or both of them are not in the network, (2) there is no link between the pairs, and (3) the pair is linked. We defined the average length of the matrix as the mean of all linked pairs, and k-th level neighbor as the pair of probes whose distance between them is k. It suggested that the number of third and fourth level neighbors, and pairs that are not connected decreased sharply. However, that the average lengths of shortest path matrices changed marginally, and that all 6 levels (from first to sixth) remain between probes of the minimized signatures and their input, suggesting that the topological properties of the PPI subsets consist of stable features, which are not changed by MFS (Additional file 10).

## Discussion

Gene signatures of large and small size could perform equally well in clinical applications. For example, the NIEHS and SAI predictors for the breast cancer endpoint E (NIEHS_BR_E_5 [982 features] and SAI_BR_E_1 [51 features] respectively) have close predictive powers (Validation MCC of both signatures is 0.748), but completely different feature sizes. The above suggests that it is probable to minimize the size of gene signatures while maintaining their predictive power. This notion is also supported by previous study that small gene signatures can perform well in discriminative analyses [23].

Biological importance can be inferred through simple similarity analyses of gene signatures for each studied endpoint on the overlapping genes. Interestingly, a number of predictive gene markers were experimentally confirmed to be related to breast cancer (Table 3). These observations are consistent with all other predictable endpoints of the MAQC-II project. For example, *CA12*, a highly correlated gene with estrogen receptor α (ERα), is robustly regulated by estrogen via ERα in breast cancer cells, and this regulation involves a distal estrogen-responsive enhancer region [24,25]. *ESR1 *encodes an estrogen receptor, a ligand-activated transcription factor composed of several domains important for hormone binding, DNA binding, and activation of transcription [25]. Besides, high levels of *MAPT *(*microtubule-associated protein tau*) mRNA expression in ER-positive breast cancer indicate an endocrine-sensitive, but chemotherapy-resistant disease. In contrast, low *tau *expression levels are associated with a subset of ER-positive cancers that have poor prognosis with tamoxifen alone and may benefit from taxane-containing chemotherapy [26]. Moreover, *GATA3 *(*GATA binding protein 3*) is reported as a breast cancer marker and is expressed almost among all ER-positive tumors [25]. Low levels of *GATA3 *are associated with invasive breast carcinomas [25]. Numerous studies, notably based on microarray data, have shown that expression of *GATA3 *is strongly and positively correlated with that of *ESR1*. The strong correlation between *ESR1 *and *GATA3 *expression in breast cancer tissues implies that *GATA3 *might cooperate with this steroid receptor to regulate breast tissue-specific hormone-responsive genes [27].

Since the minimization process can remove probes regardless of their ranking, some top-ranked probes are removed without affecting the predictive power of the model. To find out the reasons, we re-mapped the probes for two minimized signatures to corresponding genes, and no overlapping gene was found in the re-mapped list for BR_D_Model and only 4 genes overlapped in BR_E_Model. That is, probes with more than one corresponding genes were rarely observed after the minimized process. To further inquire this phenomenon, we also examined the distribution of these genes based on the pathways archived at MsigDB [28]. Although the number of genes in the signatures was small, a large number of pathways were found to be represented, and most of these pathways included only one or two genes in each pathway. Among the genes involved in multiple pathways, *CCND1, IL8, IGF1R*, and *MYB *participate in more than 40, while numerous genes involve in same pathways, e.g. *BRCA_ER_NEG, BRCA_ER_POS, STEMCELL_NEURAL_UP*, and *LEI_MYB_REGULATED_GENES*. This finding suggests that our minimized gene signatures are highly representative of multiple important pathways that may be involved in the biological processes underlying the discrimination of normal tissue from breast cancer samples. In that way, the rationale behind the phenomenon could be as follows: when some top-ranked overlapping probes are removed, the non-overlapping probes retain sufficient discriminatory power as the remaining probes could still stands for the majority of genes and pathways.

Based on the essence of feasibility for the minimized methodology and biological functions inferred under similarity analyses, we further explored the rationale for the consistency and the diversity of the gene signatures. The gene signatures generated from different teams for the same clinical outcome are different from each other, with some failing to share any gene in common. The diversity could result from the use of different feature selection methods, classification algorithms etc. Similarly, gene signatures for different clinical outcomes of same disease have been shown to exhibit little overlap between features [29-31], This observation has been attributed to the use of multiple factors, such as different datasets, feature selection methods, classification algorithms sample sizes, and patient diversity [32,33]. The diversity of patients includes environmental effects, age and sex, disease stages, and patient health. In addition, genes involved at different disease stages or with different disease subtypes could also be different. Furthermore, the assumptions (i.e. gene independence) for the statistical models used in gene marker identification do not typically hold up given the small sample sizes and complexity of gene interactions.

Despite of these complex issues, in some rare cases the predictive power of each model has been independently validated with large numbers of patients, and all have shown similar performance [32].

Models with better performance can be generated by probe redundancy reduction with the MFS process. Several factors may contribute to this significance. First, the input for MFS are not all overlapping probes but probes with a sticker criterion which can minimize the random effect and improve the predictive power of signature, the reason behind this is that different DATs have different modeling factors, which contain randomicity and are evaluated by MAQC-II [2]. Besides, during the feature selection process, numerous different statistical strategies have been applied for this purpose, but those features in a gene signature were purely selected based on statistical significances, some of them may not have any relation to the studied endpoint phenotype but somehow are correlated to the genes related to the endpoint. Those features may not have the positive contribution to the model performance but generate certain noise to interfere with the predictive ability. Our method can identify those genes and exclude them from the minimized features, eventually, lead to improve the predictive power.

MFS method generally reduces redundancies for features within gene signatures and improves the performance of the model (Table 2), which indicates the existence of consistency for the studying endpoint. Clinical applications will benefit from the gene signature reduction, since the reduced size of gene signature with similar or better performance can increase the efficiency and reduce cost. Meanwhile, most of the features remaining in the minimized gene signature tend to have a strong association with the disease and the application of those disease oriented features in diagnosis is more informative. To solve this problem, we use an MCC-robustness value (Methods) as a measurement for feature selection process and examine their biological functions through GO term analysis. However, the predictive power of newly-generated classifiers depends on the quality of training and validation datasets, as well as the collected features and the selected classification algorithm. A newly generated gene signature by MFS would never perform well if the performance of its input signatures is not good.

MFS could benefit the clinical applications of microarray technology in several ways. Firstly, it could improve the predictive power of signatures, which is a probable contribution to the implementation of personalized medicine; secondly, it minifies the number of probes in signatures, which can reduce cost for microarray's applications, and more important, it can avoid the weaknesses of large-size signatures: the insufficiency of sample, relevance among features, and the possible inaccuracy. Thirdly, the similarity analyses can disclose the consistency and diversity among signatures for a disease, which is related to the essential of the disease.

## Conclusion

Generally, our analyses of results from MAQC-II project indicate that gene signatures of both large and small sizes could perform equally well in clinical applications. In that way, it is reasonable to minimize the size of gene signatures. Besides, biological significances can be inferred through similarity analyses, the results of which are the expected consistency for multiple gene signatures, reflecting the studying endpoints. MFS was developed following this principle. As a result, new classifiers built with minimized features based on similarity analyses can reflect breast cancer-related pathways, and can always have a smaller size and a significant predictive power. No doubt, the strategy could help the microarray-based clinical applications.

## Methods

### Datasets and endpoints

Totally we analyzed two binary endpoints: pathologic complete response (endpoint D) and estrogen receptor status (endpoint E). MAQC-II provided two key datasets: training dataset [14] and validation dataset [14]. Training dataset contains 130 samples (33 positives and 97 negatives for endpoint D, 80 positives and 50 negatives for endpoint E), and validation dataset contains 100 samples (15 positives and 85 negatives for endpoint D, 61 positives and 39 negatives for endpoint E). EV1 dataset [15-17] and EV2 dataset [18] were also used for external validations. All datasets were generated using Affymetrix U133A or U133B platforms, with their research targets be endpoint D or endpoint E. More parameters for datasets are available at Additional file 11.

For new gene signatures, we utilize the classification method of support vector machine (SVM) and repeat five-fold cross validation (5F-CV) ten times, using the training dataset as an internal reference. The minimized signatures were built on the training dataset and validated on the validation dataset, and extra-validations for endpoint E were also proposed using EV1 dataset and EV2 dataset. Correspondingly, the swap models are signatures built on validation dataset and be validated on the training dataset. It is worthwhile to mention that the training and validation datasets are profiled separately in different timeframes, thus providing a real-world clinical application scenario.

### Preprocessing for the input models

Preprocessing was used as a quality control process. Some of the models were selected for our research, from 2 997 models for endpoint D and 1 196 for endpoint E models, among which informal models were excluded. However, only features of about 30 gene signatures for each endpoint are available. A null check for key attributes was also proposed, that is, an item would be discarded if any of its key attributes are absent. Models' parameters are provided as Additional file 12 and 13.

### Similarity analyses

For each endpoint, similarity analyses were performed, at the level of probe, gene and GO. Probes extracted from different signatures were compared to identify overlap at the probe level, and a unique set of probes was generated, which only contains these probes identified by at least two sources (or a sticker criterion). At the gene level, the unique set of genes was generated in a similar way. Yet, transforming probes to corresponding genes presents a challenge, as some probes have more than one corresponding gene on the Affymetrix platform. For probes that only have one corresponding gene, an Affymertix annotation file for U133A platform is used [34]. If genes that correspond to a given probe are isoenzymes or aliases, then they should be considered as one, while pseudogenes and hypothetical genes were excluded. For the remaining probes, we transformed them to its closest gene using a Bayesian-Decision Tree (Bayesian-Decision Tree document in Additional file 14.

At the GO level, GoMiner [19], a freely available graphical user interface program developed by Zeeberg et al (2003), was used to calculate the relative enrichment factor and the most involved pathways for the gene signatures. The entire gene list for Affymetrix U133A was defined as total genes for each endpoint, while genes extracted from input gene signatures were defined as subsets. GO Categories terms with p-values larger than 0.05 were excluded. The categories lists were sorted by number of genes with changing expression, and top terms of biological process, cellular component, and molecular function were selected out, respectively.

There are two supplemental similarity analyses along with the previous mentioned levels. Analysis of gene expression level is available at Local Network Model (LNM) document in Additional file 15. The classification algorithm level similarity analysis was based on analysis of three-dimensional graphs of model performances for original models and swap models (Figure 3).

Similarity analyses between endpoints D and E were also carried out at multiple levels. At the levels of probe and gene, we checked the percentage of unique probes and genes and overlapping probes and genes shared by both endpoints and the particular shares for each endpoint. Besides, mean equal test for both endpoints were proposed for normal models and swap models (Table 1). At GO level, top-ranked terms for the two endpoints were compared for all three GO categories to see if there is any term shared by both endpoints. To further process the similarity analyses between both endpoints, we proposed cross-prediction between these two endpoints.

### Generating a new gene signature by MFS

A Java console program called Minimize Feature's Size (MFS) was implemented based on WEKA's application program interface [35]. It took three inputs, (1) an ARFF file (the default file format of WEKA), from which we obtained the feature list, expression profile data and the endpoint status, (2) a criteria value for MCC, and (3) the classifier's name. Three major goals were devised for MFS: minimal feature size, maximal internal MCC value, and maximal external MCC value.

We merged features of the input gene signatures to a unique weighted set. For each gene signature, we assigned the probes' weight with MCC-robustness values, as an un-weighted method do not considers the quality of probes, on a hypothesis that qualities of probes of a well-performed gene signature are better than those of bad-performed ones. MFS can accept multiple inputs, from multiple gene signatures to the probes of a single gene signature or probes obtained by using LNM (details are available at MFS document in Additional file 16.

During the analyses, we constructed the gene signature, eliminating genes that do not provide substantial additional information [4], or that are redundant based on similarity analyses. Fundamentally, MFS is a backward stepwise elimination [36]: For each iteration, we calculated the starting MCC value of the gene signature; Next, for each feature in the given gene signature, MFS calculated a new MCC value without the feature. If the new MCC value was less than the old one, MFS restored the previous conditions and omitted this probe; otherwise, it moved to the next probe. At the end of each iteration, MFS checked the MCC value. If it was greater than the given criteria value, MFS produced an intermediate result and then went on to the next iteration. If the size of the gene signature no longer decreased after an iteration or if the size was less or equal than a given criterion (default 5), MFS terminated the process and recorded the model marked as 'best'. Note that the 'best' model was saved whether its MCC value was larger than the given cutoff or not. It is also possible to use other indices to do the fitting process (see **Statistical analysis**).

Array-generation based gene centering (AGC) of the datasets was used as an optional process in MFS to remove background noise [37]. External validation of newly produced ARFF files would be executed to check the stability of the new gene signatures. Only gene signatures that performed well both in internal and external validations were retained.

### Statistical analysis

We used the MCC [38] as the index for classifier predictive power. TP = true positive, TN = true negative, FP = false positive, FN = false negative.

$$\text{MCC}=\frac{\text{TP}\times \text{TN}-\text{FP}\times \text{FN}}{\sqrt{\left(\text{TP}+\text{FP}\right)\left(\text{TP}+\text{FN}\right)}\left(\text{TN}+\text{FP}\right)\;\;\left(\text{TN}+\text{FN}\right)}$$

An MCC-robustness value is used to measure the predictive power and its stability of a classifier, a measurement index for top probes' selection in the MFS. A well-performed classifier would have a larger MCC value and a smaller standard deviation. Once the MCC-robustness value approaches infinity it is assigned the smallest weight.

$$\text{MCC}-\text{robustness}=\frac{\bar{\text{MCC}}}{\sigma_{MCC}}$$

Other indices that were used in the MFS fitting process besides MCC:

$$\text{index1}=\frac{\text{TP}\times \text{TN}}{\left(\text{TP}+\text{FP}\right)\left(\text{TN}+\text{FP}\right)}$$

$$\text{index2}=\frac{\text{TN}}{\text{TN}+\text{FP}}+\frac{\text{TP}}{\text{TP}+\text{FP}}$$

## Authors' contributions

TS, CC and JW conceived and designed the study. CC and CZ performed the programming tasks. JW finished the statistical designs and analyzed the data. CC and JW drafted the manuscript. TS, WT, LP, YD and WFS have been involved in revising the manuscript. PRB provided an independent assessment for comments and suggestions of WT. JF offered a supplementary analysis for the manuscript. All authors have read and approved the final manuscript.

## Competing interests

The authors declare that they have no competing interests.

^※^**Disclaimer: **The views presented in this article do not necessarily reflect those of the U.S. Food and Drug Administration.

## Supplementary Material

Additional file 1 **Endpoint D probe level overlap matrix**. Click here for file

Additional file 2 **Endpoint E probe level overlap matrix**. Click here for file

Additional file 3 **Top probes (Similarity Analysis)**. Click here for file

Additional file 4 **Top 500 probes' differentially expressed probes analysis**. Click here for file

Additional file 5 **Further analyses at probe level**. Click here for file

Additional file 6 **Internal and external validation for endpoint E**. Click here for file

Additional file 7 **Heatmaps for original models and swap models on training dataset and validation dataset**. Click here for file

Additional file 8 **Top genes (Similarity Analysis)**. Click here for file

Additional file 9 **GO term enrichment for overlapping genes (Similarity Analysis)**. Click here for file

Additional file 10 **All pairs shortest path matrix counting for features before and after MFS**. Click here for file

Additional file 11 **Datasets**. Click here for file

Additional file 12 **All pCR models as the input of CAS_BR_D_4**. Click here for file

Additional file 13 **All ER models as the input of CAS_BR_E_15**. Click here for file

Additional file 14 **Bayesian-Decision Tree Method**. Click here for file

Additional file 15 **Development of the Local Network Model (LNM) tool**. Click here for file

Additional file 16 **MFS inputs**. Click here for file

Additional file 17 **Features of the CAS_BR_D_4 and CAS_BR_E_15**. Click here for file
