# Supplementary material for: Maximizing biomarker discovery by minimizing gene signatures
Source: BMC Genomics. 2011 Dec 23;12(Suppl 5):S6. doi: 10.1186/1471-2164-12-S5-S6 (PMC3287502; doi:10.1186/1471-2164-12-S5-S6)
Supplement: Additional file 1 — Endpoint D probe level overlap matrix. [file 1471-2164-12-S5-S6-S1.doc]

**Table S1: Endpoint D probe level overlap matrix**

| Matrix | CAS_BR_D_1 | CAS_BR_D_2 | CBC_BR_D_1 | CBC_BR_D_2 | CDRH_BR_D_4 | DKFZ_BR_D_1 | FBK_BR_D_1 | FBK_BR_D_2 | GSK_BR_D_1 | GT_BR_D_4 | NIEHS_BR_D_8 | NWU_BR_D_13 | SDSU_BR_D_2 | Spheromics_BR_D_1 | UIUC_BR_D_1 | USM_BR_D_1 | ABT_BR_D_1 | CIPF_BR_D_1 | GeneGo_BR_D_3 | JHSPH_BR_D_2 | NCTR_BR_D_1 | NWU_BR_D_1 | Roche_BR_D_1 | SAI_BR_D_1 | SAS_BR_D_M23_PLS_023 | SDSU_BR_D_1 | Tsinghua_BR_D_6 | ZJU_BR_D_1 | **CAS_BR_D_4** |
| --- | --- | --- | --- | --- | --- | --- | --- | --- | --- | --- | --- | --- | --- | --- | --- | --- | --- | --- | --- | --- | --- | --- | --- | --- | --- | --- | --- | --- | --- |
| CAS_BR_D_1 | 11 |  |  |  |  |  |  |  |  |  |  |  |  |  |  |  |  |  |  |  |  |  |  |  |  |  |  |  |  |
| CAS_BR_D_2 | 1 | 10 |  |  |  |  |  |  |  |  |  |  |  |  |  |  |  |  |  |  |  |  |  |  |  |  |  |  |  |
| CBC_BR_D_1 | 0 | 0 | 22 |  |  |  |  |  |  |  |  |  |  |  |  |  |  |  |  |  |  |  |  |  |  |  |  |  |  |
| CBC_BR_D_2 | 2 | 1 | 22 | 38 |  |  |  |  |  |  |  |  |  |  |  |  |  |  |  |  |  |  |  |  |  |  |  |  |  |
| CDRH_BR_D_4 | 0 | 0 | 12 | 13 | 25 |  |  |  |  |  |  |  |  |  |  |  |  |  |  |  |  |  |  |  |  |  |  |  |  |
| DKFZ_BR_D_1 | 0 | 0 | 2 | 2 | 3 | 13 |  |  |  |  |  |  |  |  |  |  |  |  |  |  |  |  |  |  |  |  |  |  |  |
| FBK_BR_D_1 | 1 | 0 | 3 | 5 | 1 | 1 | 10 |  |  |  |  |  |  |  |  |  |  |  |  |  |  |  |  |  |  |  |  |  |  |
| FBK_BR_D_2 | 2 | 1 | 17 | 22 | 15 | 3 | 9 | 100 |  |  |  |  |  |  |  |  |  |  |  |  |  |  |  |  |  |  |  |  |  |
| GSK_BR_D_1 | 0 | 0 | 3 | 3 | 1 | 1 | 3 | 3 | 3 |  |  |  |  |  |  |  |  |  |  |  |  |  |  |  |  |  |  |  |  |
| GT_BR_D_4 | 0 | 0 | 0 | 0 | 0 | 0 | 0 | 0 | 0 | 10 |  |  |  |  |  |  |  |  |  |  |  |  |  |  |  |  |  |  |  |
| NIEHS_BR_D_8 | 4 | 1 | 16 | 24 | 13 | 5 | 9 | 30 | 3 | 0 | 68 |  |  |  |  |  |  |  |  |  |  |  |  |  |  |  |  |  |  |
| NWU_BR_D_13 | 0 | 0 | 8 | 8 | 7 | 2 | 2 | 6 | 2 | 0 | 6 | 15 |  |  |  |  |  |  |  |  |  |  |  |  |  |  |  |  |  |
| SDSU_BR_D_2 | 2 | 1 | 14 | 19 | 14 | 9 | 4 | 28 | 3 | 0 | 28 | 5 | 50 |  |  |  |  |  |  |  |  |  |  |  |  |  |  |  |  |
| Spheromics_BR_D_1 | 6 | 2 | 13 | 19 | 15 | 11 | 10 | 52 | 3 | 0 | 33 | 6 | 36 | 206 |  |  |  |  |  |  |  |  |  |  |  |  |  |  |  |
| UIUC_BR_D_1 | 1 | 0 | 6 | 8 | 5 | 3 | 1 | 3 | 1 | 0 | 5 | 3 | 8 | 6 | 18 |  |  |  |  |  |  |  |  |  |  |  |  |  |  |
| USM_BR_D_1 | 2 | 2 | 13 | 16 | 12 | 5 | 2 | 20 | 2 | 0 | 27 | 6 | 21 | 19 | 5 | 50 |  |  |  |  |  |  |  |  |  |  |  |  |  |
| ABT_BR_D_1 | 0 | 0 | 5 | 5 | 2 | 2 | 2 | 4 | 2 | 0 | 5 | 1 | 6 | 5 | 3 | 4 | 6 |  |  |  |  |  |  |  |  |  |  |  |  |
| CIPF_BR_D_1 | 2 | 2 | 20 | 31 | 17 | 4 | 5 | 25 | 3 | 0 | 31 | 13 | 28 | 28 | 16 | 28 | 6 | 90 |  |  |  |  |  |  |  |  |  |  |  |
| GeneGo_BR_D_3 | 1 | 1 | 4 | 4 | 3 | 1 | 2 | 5 | 1 | 0 | 6 | 1 | 7 | 5 | 4 | 2 | 4 | 6 | 9 |  |  |  |  |  |  |  |  |  |  |
| JHSPH_BR_D_2 | 0 | 0 | 0 | 0 | 0 | 0 | 0 | 0 | 0 | 0 | 0 | 0 | 0 | 0 | 0 | 0 | 0 | 0 | 0 | 24 |  |  |  |  |  |  |  |  |  |
| NCTR_BR_D_1 | 1 | 0 | 1 | 2 | 0 | 0 | 4 | 5 | 1 | 0 | 4 | 0 | 1 | 5 | 0 | 1 | 1 | 1 | 0 | 0 | 5 |  |  |  |  |  |  |  |  |
| NWU_BR_D_1 | 5 | 8 | 21 | 36 | 24 | 13 | 10 | 81 | 3 | 4 | 60 | 15 | 48 | 151 | 17 | 47 | 6 | 88 | 9 | 2 | 5 | 1622 |  |  |  |  |  |  |  |
| Roche_BR_D_1 | 0 | 0 | 3 | 4 | 3 | 0 | 0 | 3 | 0 | 0 | 5 | 2 | 3 | 3 | 2 | 2 | 1 | 4 | 2 | 0 | 0 | 10 | 10 |  |  |  |  |  |  |
| SAI_BR_D_1 | 2 | 3 | 14 | 17 | 14 | 3 | 3 | 16 | 2 | 0 | 22 | 7 | 22 | 18 | 13 | 17 | 5 | 37 | 6 | 0 | 0 | 62 | 4 | 68 |  |  |  |  |  |
| SAS_BR_D_M23_PLS_023 | 3 | 5 | 18 | 24 | 21 | 12 | 5 | 32 | 3 | 0 | 28 | 9 | 42 | 47 | 14 | 27 | 6 | 53 | 8 | 0 | 2 | 114 | 3 | 35 | 120 |  |  |  |  |
| SDSU_BR_D_1 | 2 | 1 | 11 | 14 | 10 | 5 | 4 | 17 | 3 | 0 | 17 | 4 | 30 | 25 | 6 | 13 | 5 | 19 | 6 | 0 | 1 | 29 | 2 | 15 | 27 | 30 |  |  |  |
| Tsinghua_BR_D_6 | 0 | 0 | 7 | 10 | 5 | 3 | 5 | 14 | 2 | 0 | 18 | 4 | 16 | 23 | 5 | 2 | 3 | 11 | 5 | 0 | 1 | 35 | 5 | 14 | 15 | 11 | 44 |  |  |
| ZJU_BR_D_1 | 1 | 0 | 0 | 0 | 1 | 1 | 1 | 15 | 0 | 0 | 8 | 0 | 4 | 21 | 0 | 1 | 0 | 0 | 0 | 0 | 0 | 34 | 0 | 1 | 4 | 3 | 8 | 40 |  |
| **CAS_BR_D_4** | 1 | 3 | 5 | 10 | 7 | 2 | 1 | 12 | 0 | 0 | 11 | 4 | 10 | 15 | 7 | 12 | 0 | 21 | 0 | 0 | 0 | 31 | 3 | 13 | 22 | 7 | 5 | 6 | 32 |

Some gene signatures have overlaps within almost all of other gene signatures. GT_BR_D_4 shares few overlaps with others and has a poor performance. MFS would move most of features in the model as only probes appeared twice or more can be selected.
