# Supplementary material for: Maximizing biomarker discovery by minimizing gene signatures
Source: BMC Genomics. 2011 Dec 23;12(Suppl 5):S6. doi: 10.1186/1471-2164-12-S5-S6 (PMC3287502; doi:10.1186/1471-2164-12-S5-S6)
Supplement: Additional file 2 — Endpoint E probe level overlap matrix. [file 1471-2164-12-S5-S6-S2.doc]

**Table S2: Endpoint E probe level overlap matrix**

| Matrix | CAS_BR_E_1 | CAS_BR_E_2 | CBC_BR_E_1 | CBC_BR_E_2 | DKFZ_BR_E_1 | FBK_BR_E_1 | GSK_BR_E_1 | GT_BR_E_2 | NIEHS_BR_E_5 | NWU_BR_E_1 | SDSU_BR_E_1 | UIUC_BR_E_1 | CIPF_BR_E_1 | GeneGo_BR_E_2 | JHSPH_BR_E_2 | NCTR_BR_E_1 | SAI_BR_E_1 | SAS_BR_E_M03_LR_015 | Tsinghua_BR_E_50 | USM_BR_E_1 | ZJU_BR_E_1 | ABT_BR_E_1 | CAS_BR_E_9 | **CAS_BR_E_15** |
| --- | --- | --- | --- | --- | --- | --- | --- | --- | --- | --- | --- | --- | --- | --- | --- | --- | --- | --- | --- | --- | --- | --- | --- | --- |
| CAS_BR_E_1 | 15 |  |  |  |  |  |  |  |  |  |  |  |  |  |  |  |  |  |  |  |  |  |  |  |
| CAS_BR_E_2 | 0 | 10 |  |  |  |  |  |  |  |  |  |  |  |  |  |  |  |  |  |  |  |  |  |  |
| CBC_BR_E_1 | 3 | 0 | 27 |  |  |  |  |  |  |  |  |  |  |  |  |  |  |  |  |  |  |  |  |  |
| CBC_BR_E_2 | 3 | 0 | 27 | 50 |  |  |  |  |  |  |  |  |  |  |  |  |  |  |  |  |  |  |  |  |
| DKFZ_BR_E_1 | 3 | 0 | 6 | 6 | 6 |  |  |  |  |  |  |  |  |  |  |  |  |  |  |  |  |  |  |  |
| FBK_BR_E_1 | 3 | 0 | 12 | 14 | 4 | 15 |  |  |  |  |  |  |  |  |  |  |  |  |  |  |  |  |  |  |
| GSK_BR_E_1 | 4 | 2 | 27 | 49 | 6 | 15 | 316 |  |  |  |  |  |  |  |  |  |  |  |  |  |  |  |  |  |
| GT_BR_E_2 | 0 | 0 | 1 | 1 | 1 | 1 | 2 | 10 |  |  |  |  |  |  |  |  |  |  |  |  |  |  |  |  |
| NIEHS_BR_E_5 | 3 | 7 | 27 | 50 | 6 | 15 | 238 | 5 | 984 |  |  |  |  |  |  |  |  |  |  |  |  |  |  |  |
| NWU_BR_E_1 | 2 | 0 | 7 | 13 | 3 | 4 | 49 | 0 | 59 | 141 |  |  |  |  |  |  |  |  |  |  |  |  |  |  |
| SDSU_BR_E_1 | 3 | 0 | 25 | 37 | 6 | 13 | 95 | 2 | 99 | 23 | 100 |  |  |  |  |  |  |  |  |  |  |  |  |  |
| UIUC_BR_E_1 | 3 | 0 | 17 | 21 | 6 | 9 | 21 | 1 | 23 | 8 | 17 | 23 |  |  |  |  |  |  |  |  |  |  |  |  |
| CIPF_BR_E_1 | 3 | 0 | 22 | 27 | 6 | 10 | 27 | 1 | 30 | 7 | 21 | 20 | 30 |  |  |  |  |  |  |  |  |  |  |  |
| GeneGo_BR_E_2 | 2 | 0 | 6 | 6 | 2 | 5 | 7 | 0 | 7 | 2 | 6 | 5 | 6 | 7 |  |  |  |  |  |  |  |  |  |  |
| JHSPH_BR_E_2 | 0 | 0 | 0 | 0 | 0 | 0 | 0 | 0 | 0 | 0 | 0 | 0 | 0 | 0 | 22 |  |  |  |  |  |  |  |  |  |
| NCTR_BR_E_1 | 1 | 0 | 3 | 4 | 1 | 4 | 5 | 0 | 5 | 2 | 5 | 1 | 1 | 1 | 0 | 5 |  |  |  |  |  |  |  |  |
| SAI_BR_E_1 | 3 | 0 | 27 | 49 | 6 | 14 | 51 | 1 | 51 | 12 | 39 | 21 | 27 | 6 | 0 | 4 | 51 |  |  |  |  |  |  |  |
| SAS_BR_E_M03_LR_015 | 2 | 0 | 8 | 11 | 5 | 5 | 12 | 1 | 14 | 4 | 11 | 7 | 8 | 2 | 0 | 2 | 11 | 14 |  |  |  |  |  |  |
| Tsinghua_BR_E_50 | 5 | 5 | 24 | 46 | 6 | 13 | 165 | 5 | 492 | 52 | 89 | 23 | 27 | 6 | 0 | 5 | 47 | 14 | 1150 |  |  |  |  |  |
| USM_BR_E_1 | 2 | 0 | 14 | 18 | 4 | 8 | 26 | 1 | 30 | 6 | 19 | 13 | 14 | 4 | 0 | 2 | 19 | 6 | 28 | 30 |  |  |  |  |
| ZJU_BR_E_1 | 1 | 1 | 0 | 2 | 0 | 2 | 118 | 0 | 129 | 34 | 45 | 0 | 0 | 0 | 0 | 1 | 3 | 0 | 68 | 3 | 145 |  |  |  |
| ABT_BR_E_1 | 1 | 0 | 5 | 6 | 1 | 3 | 6 | 0 | 6 | 1 | 5 | 4 | 5 | 1 | 0 | 1 | 6 | 2 | 5 | 3 | 0 | 6 |  |  |
| CAS_BR_E_9 | 2 | 2 | 18 | 30 | 2 | 10 | 54 | 1 | 58 | 9 | 39 | 11 | 18 | 5 | 0 | 4 | 32 | 6 | 49 | 9 | 21 | 6 | 58 |  |
| **CAS_BR_E_15** | 2 | 0 | 11 | 18 | 3 | 7 | 55 | 1 | 50 | 18 | 28 | 10 | 11 | 2 | 0 | 2 | 19 | 6 | 44 | 9 | 36 | 3 | 19 | 55 |
