# Supplementary material for: Maximizing biomarker discovery by minimizing gene signatures
Source: BMC Genomics. 2011 Dec 23;12(Suppl 5):S6. doi: 10.1186/1471-2164-12-S5-S6 (PMC3287502; doi:10.1186/1471-2164-12-S5-S6)
Supplement: Additional file 3 — Top probes (Similarity Analysis). [file 1471-2164-12-S5-S6-S3.doc]

**Table S3: Top probes (Similarity Analysis)**

| Endpoint D | | Endpoint E | | Endpoint D | | Endpoint E | | Endpoint D | | Endpoint E | |
| --- | --- | --- | --- | --- | --- | --- | --- | --- | --- | --- | --- |
| Probe | Time points | Probe | Time points | Probe | Time points | Probe | Time points | Probe | Time points | Probe | Time points |
| 205225_at | 21 | 205225_at | 19 | 210735_s_at | 10 | 215729_s_at | 10 | 209604_s_at | 7 | 206754_s_at | 7 |
| 204508_s_at | 15 | 212956_at | 15 | 218807_at | 10 | 201983_s_at | 9 | 209842_at | 7 | 207038_at | 7 |
| 206401_s_at | 15 | 209604_s_at | 14 | 220625_s_at | 10 | 203256_at | 9 | 213338_at | 7 | 209460_at | 7 |
| 209173_at | 15 | 214164_x_at | 14 | 208711_s_at | 9 | 203628_at | 9 | 214440_at | 7 | 210085_s_at | 7 |
| 205696_s_at | 14 | 214440_at | 14 | 208712_at | 9 | 204863_s_at | 9 | 215729_s_at | 7 | 212196_at | 7 |
| 215867_x_at | 14 | 217838_s_at | 14 | 210147_at | 9 | 212195_at | 9 | 218211_s_at | 7 | 218211_s_at | 7 |
| 201508_at | 13 | 202088_at | 13 | 212956_at | 9 | 218195_at | 9 | 220559_at | 7 | 218807_at | 7 |
| 203963_at | 13 | 209602_s_at | 13 | 219051_x_at | 9 | 221765_at | 9 | 221872_at | 7 | 203438_at | 6 |
| 214164_x_at | 13 | 209603_at | 13 | 204822_at | 8 | 201508_at | 8 | 203438_at | 6 | 203571_s_at | 6 |
| 203628_at | 12 | 215867_x_at | 13 | 205347_s_at | 8 | 204623_at | 8 | 204304_s_at | 6 | 203928_x_at | 6 |
| 203928_x_at | 12 | 202089_s_at | 12 | 205354_at | 8 | 209459_s_at | 8 | 204623_at | 6 | 203929_s_at | 6 |
| 204825_at | 12 | 203963_at | 12 | 206392_s_at | 8 | 211000_s_at | 8 | 204914_s_at | 6 | 204304_s_at | 6 |
| 209603_at | 12 | 204508_s_at | 11 | 206754_s_at | 8 | 211712_s_at | 8 | 209459_s_at | 6 | 204667_at | 6 |
| 213134_x_at | 12 | 210735_s_at | 11 | 209791_at | 8 | 218532_s_at | 8 | 209602_s_at | 6 | 205066_s_at | 6 |
| 203929_s_at | 11 | 205009_at | 10 | 218976_at | 8 | 218976_at | 8 | 212190_at | 6 | 205472_s_at | 6 |
| 213564_x_at | 11 | 205696_s_at | 10 | 203139_at | 7 | 204914_s_at | 7 | 212195_at | 6 | 205569_at | 6 |
| 219197_s_at | 11 | 209173_at | 10 | 204667_at | 7 | 205186_at | 7 | 212960_at | 6 | 206373_at | 6 |
| 201030_x_at | 10 | 211233_x_at | 10 | 205229_s_at | 7 | 205862_at | 7 | 216092_s_at | 6 | 206392_s_at | 6 |
| 205548_s_at | 10 | 212960_at | 10 | 208103_s_at | 7 | 206391_at | 7 | 217028_at | 6 | 211235_s_at | 6 |
| 205862_at | 10 | 215552_s_at | 10 | 209290_s_at | 7 | 206401_s_at | 7 | 217190_x_at | 6 | 212148_at | 6 |
| Endpoint D | | Endpoint E | | Endpoint D | | Endpoint E | |  | | | |
| Probe | Time points | Probe | Time points | Probe | Time points | Probe | Time points |  | | | |
| 218856_at | 6 | 212496_s_at | 6 | 202870_s_at | 4 | 205714_s_at | 5 |  | | | |
| 219497_s_at | 6 | 212771_at | 6 | 203256_at | 4 | 205734_s_at | 5 |  | | | |
| 220624_s_at | 6 | 213712_at | 6 | 203476_at | 4 | 207030_s_at | 5 |  | | | |
| 201755_at | 5 | 219197_s_at | 6 | 203571_s_at | 4 | 207076_s_at | 5 |  | | | |
| 202345_s_at | 5 | 220414_at | 6 | 203625_x_at | 4 | 209016_s_at | 5 |  | | | |
| 202641_at | 5 | 221872_at | 6 | 203627_at | 4 | 209289_at | 5 |  | | | |
| 203693_s_at | 5 | 202134_s_at | 5 | 203789_s_at | 4 | 209291_at | 5 |  | | | |
| 203930_s_at | 5 | 202341_s_at | 5 | 204750_s_at | 4 | 209373_at | 5 |  | | | |
| 205009_at | 5 | 202342_s_at | 5 | 204863_s_at | 4 | 209443_at | 5 |  | | | |
| 205044_at | 5 | 202504_at | 5 | 204913_s_at | 4 | 209791_at | 5 |  | | | |
| 206391_at | 5 | 203021_at | 5 | 204915_s_at | 4 | 210652_s_at | 5 |  | | | |
| 208358_s_at | 5 | 203637_s_at | 5 | 204962_s_at | 4 | 211002_s_at | 5 |  | | | |
| 213060_s_at | 5 | 203687_at | 5 | 205029_s_at | 4 | 211421_s_at | 5 |  | | | |
| 213527_s_at | 5 | 204533_at | 5 | 205440_s_at | 4 | 212190_at | 5 |  | | | |
| 218483_s_at | 5 | 204537_s_at | 5 | 206373_at | 4 | 213201_s_at | 5 |  | | | |
| 220540_at | 5 | 204750_s_at | 5 | 206999_at | 4 | 213338_at | 5 |  | | | |
| 201976_s_at | 4 | 204798_at | 5 | 207030_s_at | 4 | 218806_s_at | 5 |  | | | |
| 202088_at | 4 | 205044_at | 5 | 209366_x_at | 4 | 219010_at | 5 |  | | | |
| 202089_s_at | 4 | 205354_at | 5 | 209460_at | 4 | 219497_s_at | 5 |  | | | |
| 202204_s_at | 4 | 205440_s_at | 5 | 209642_at | 4 | 220540_at | 5 |  | | | |
