# Supplementary material for: Maximizing biomarker discovery by minimizing gene signatures
Source: BMC Genomics. 2011 Dec 23;12(Suppl 5):S6. doi: 10.1186/1471-2164-12-S5-S6 (PMC3287502; doi:10.1186/1471-2164-12-S5-S6)

**Figure S1: Top500 probes’ differentially expressed probes analysis**


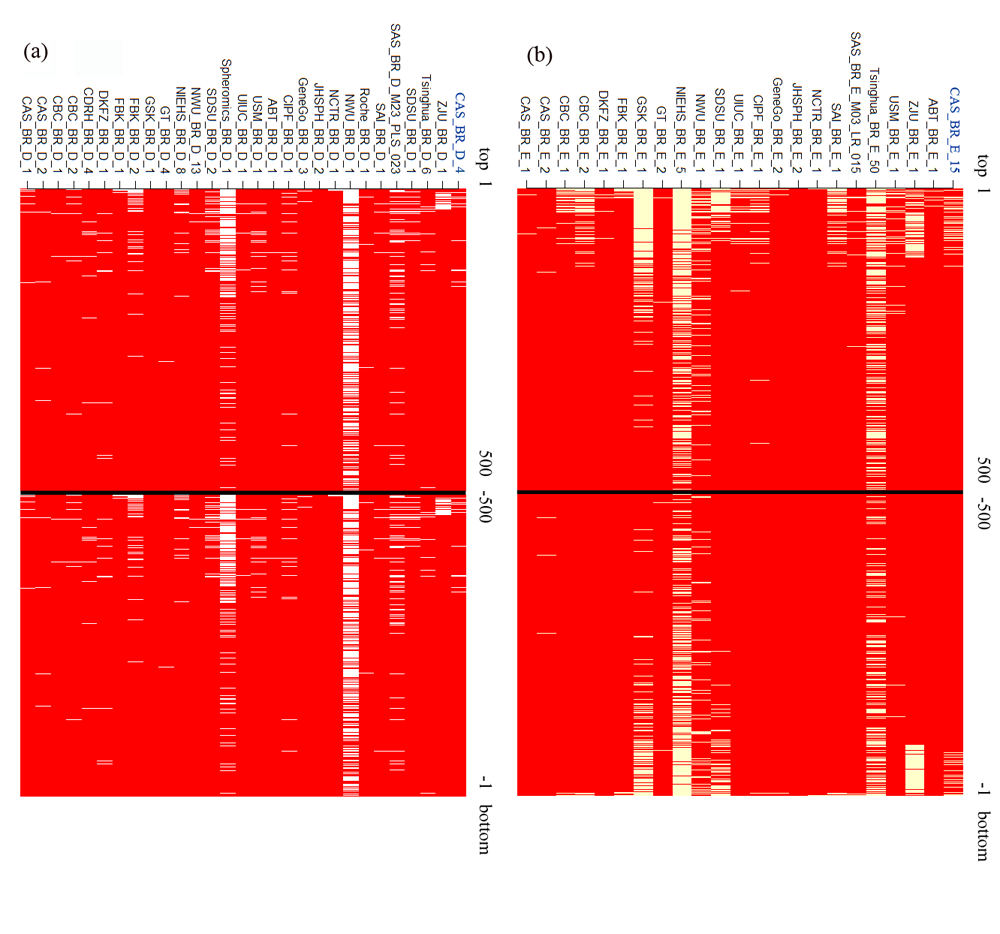


1. Endpoint D models; b) Endpoint E models.

Probes for each model were sorted according to fold-change values. White bands indicate probe positions. Two sub-graphs, corresponding to the top 500 and bottom 500 are demarcated by a black line. Our models appear in blue. The full image for the two endpoints MAQC-II models is available as Supplementary Figure S4 and S5.

**Figure S2: Endpoint D fold-change image for all probes**


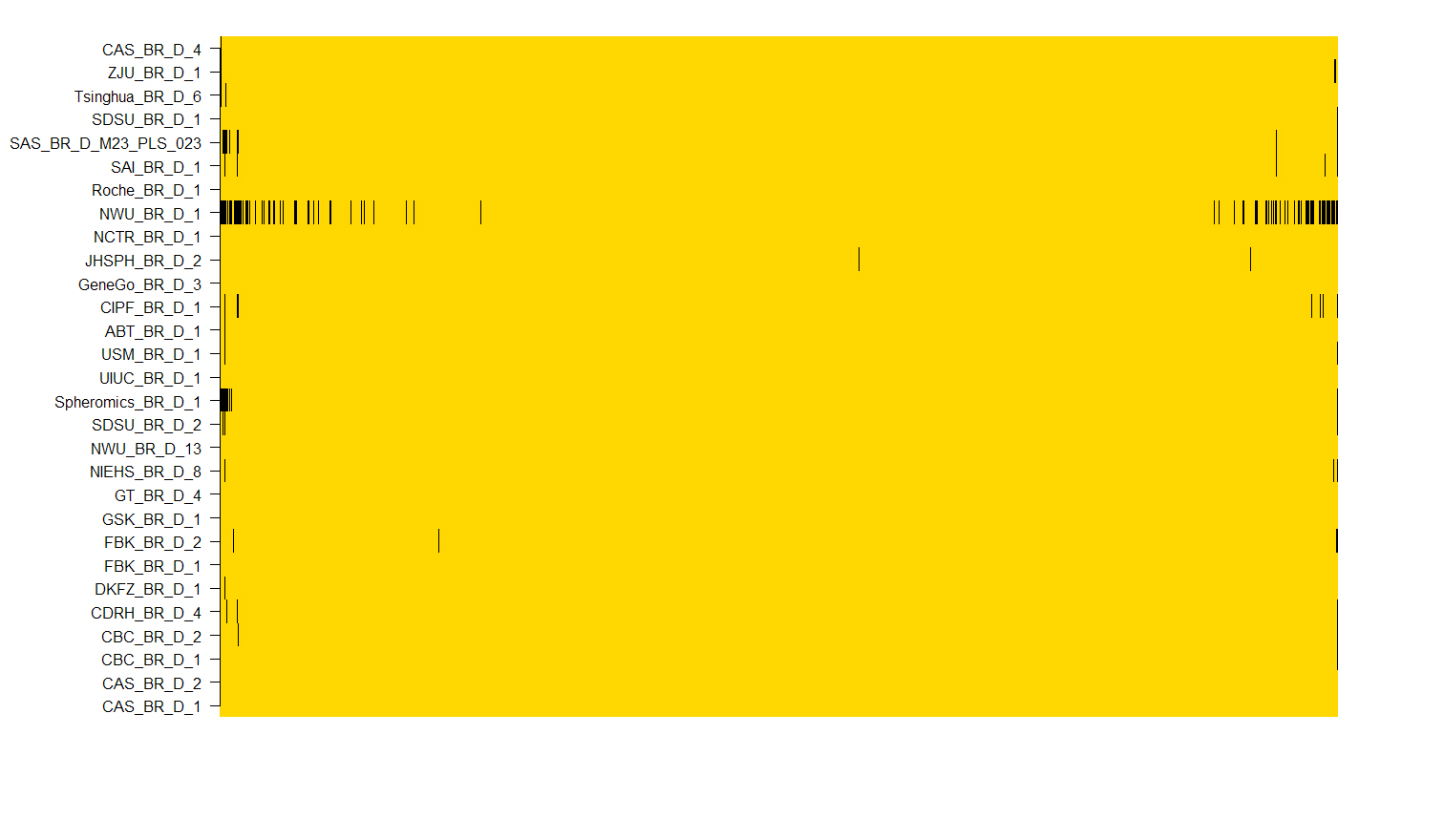


**Figure S3: Endpoint E fold-change image for all probes**


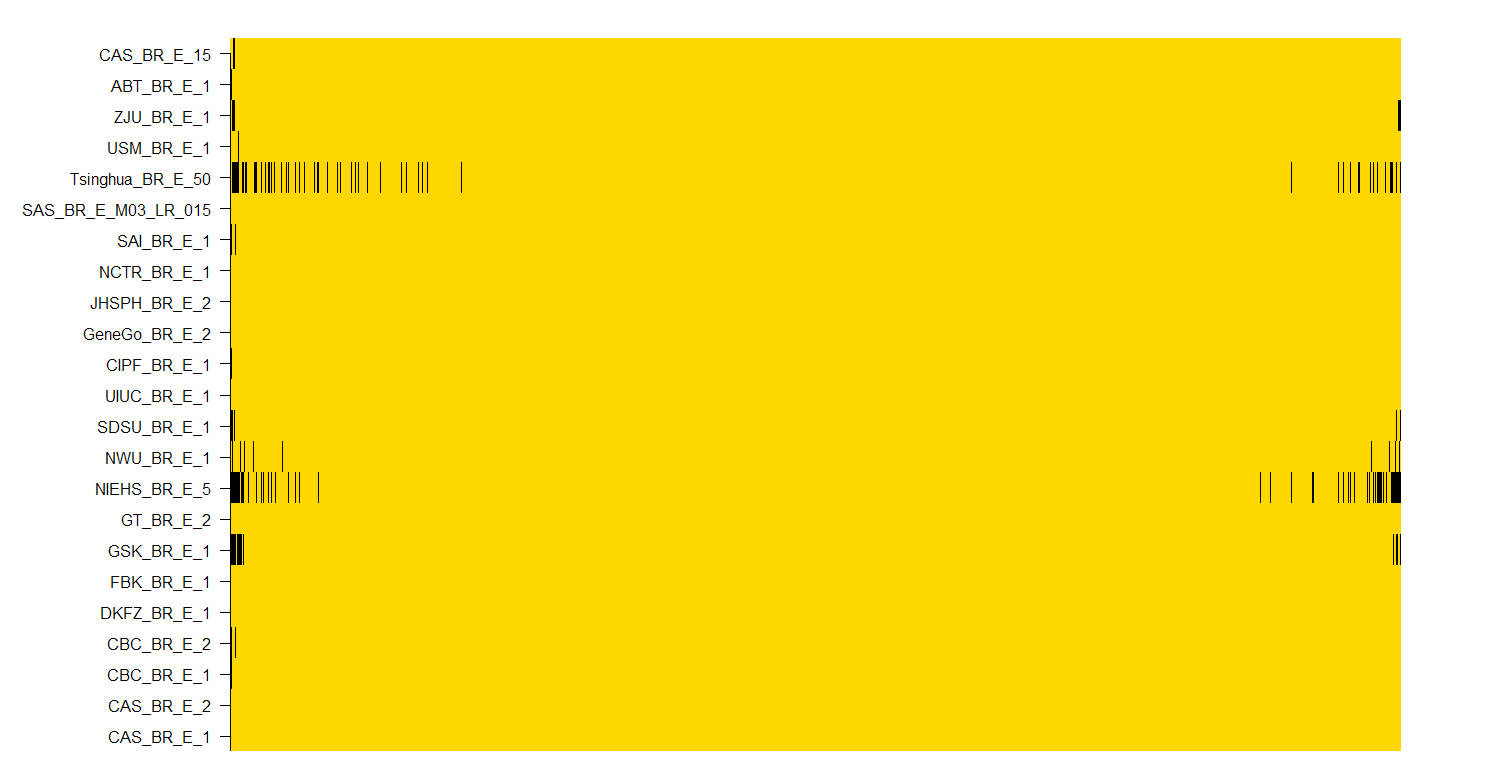

Supplement: Additional file 4 — Top 500 probes' differentially expressed probes analysis. [file 1471-2164-12-S5-S6-S4.doc]
