# Supplementary material for: Maximizing biomarker discovery by minimizing gene signatures
Source: BMC Genomics. 2011 Dec 23;12(Suppl 5):S6. doi: 10.1186/1471-2164-12-S5-S6 (PMC3287502; doi:10.1186/1471-2164-12-S5-S6)
Supplement: Additional file 5 — Further analyses at probe level. [file 1471-2164-12-S5-S6-S5.doc]

**Document S4: Further analyses at probeset level**


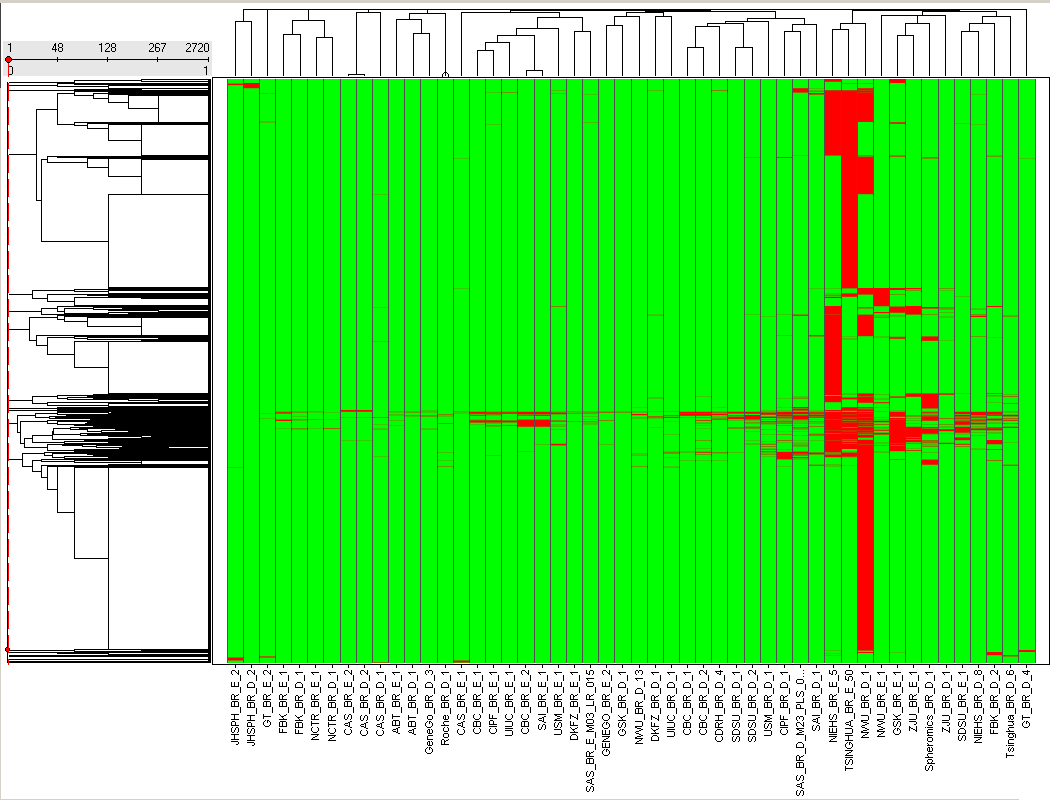


For both endpoints of breast cancer, all probesets were clustered based on Tanimoto similarity. Some probes were found in one or more models (red => found in model, green => not)


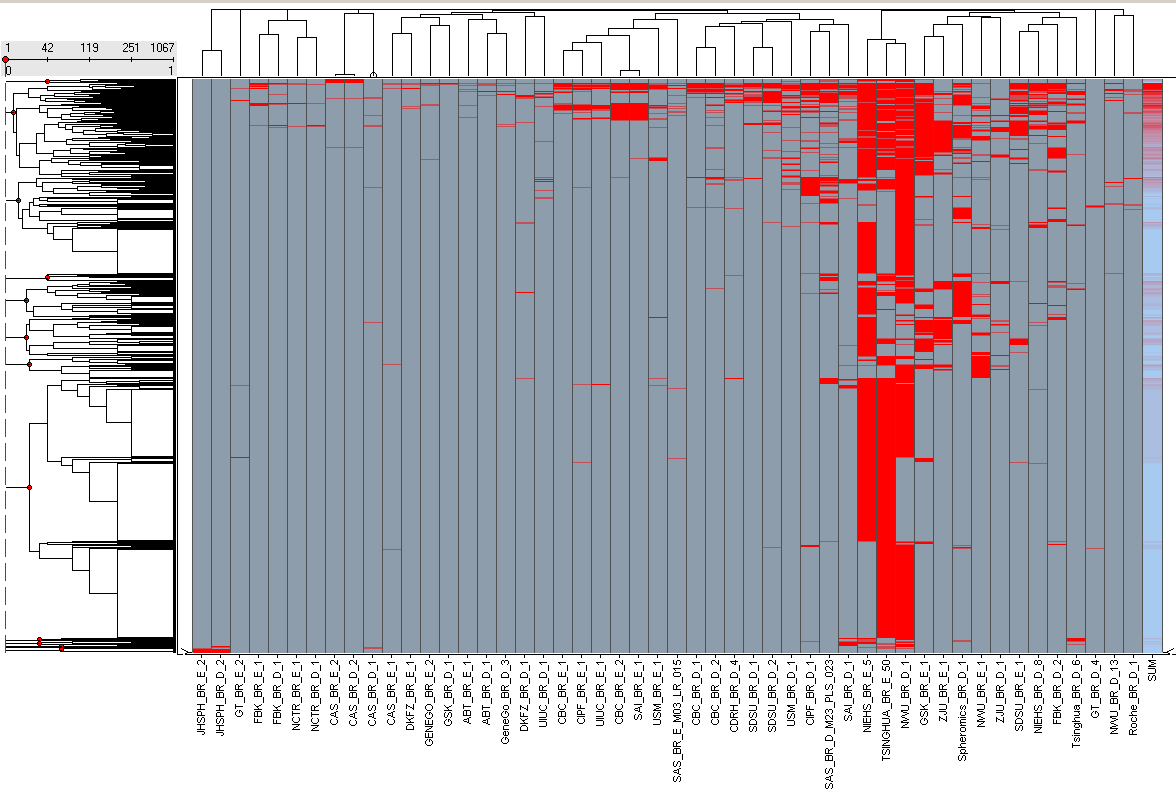


This figure illuminates probesets which were found in two or models for both D and E endpoints. Column at the right is colored by number of models containing the probeset (2 = light blue, red = 20 or more)


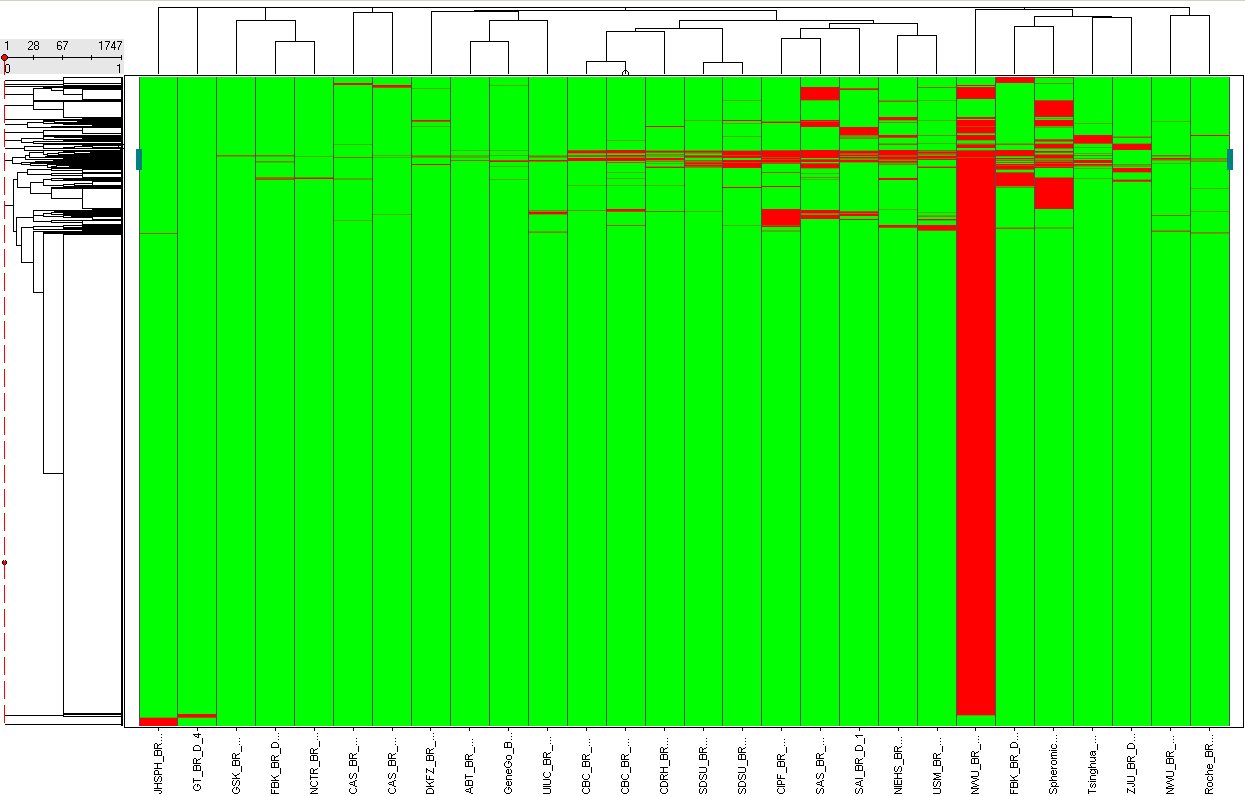

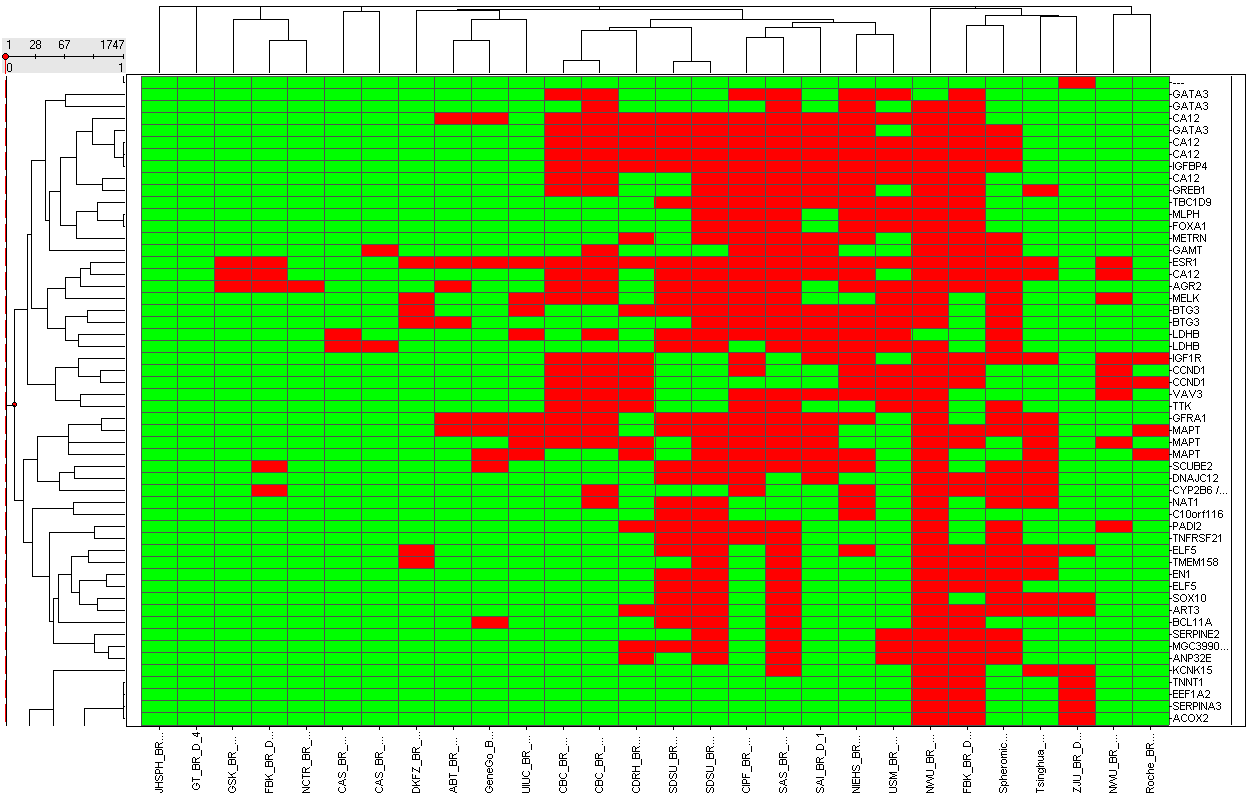


These two figures are heatmaps for endpoint D. The left one is the heatmap for all of the probes, and the right one is highlighted in top heatmap (Red => Probesets found in one or more models for endpoint D, Green => not).


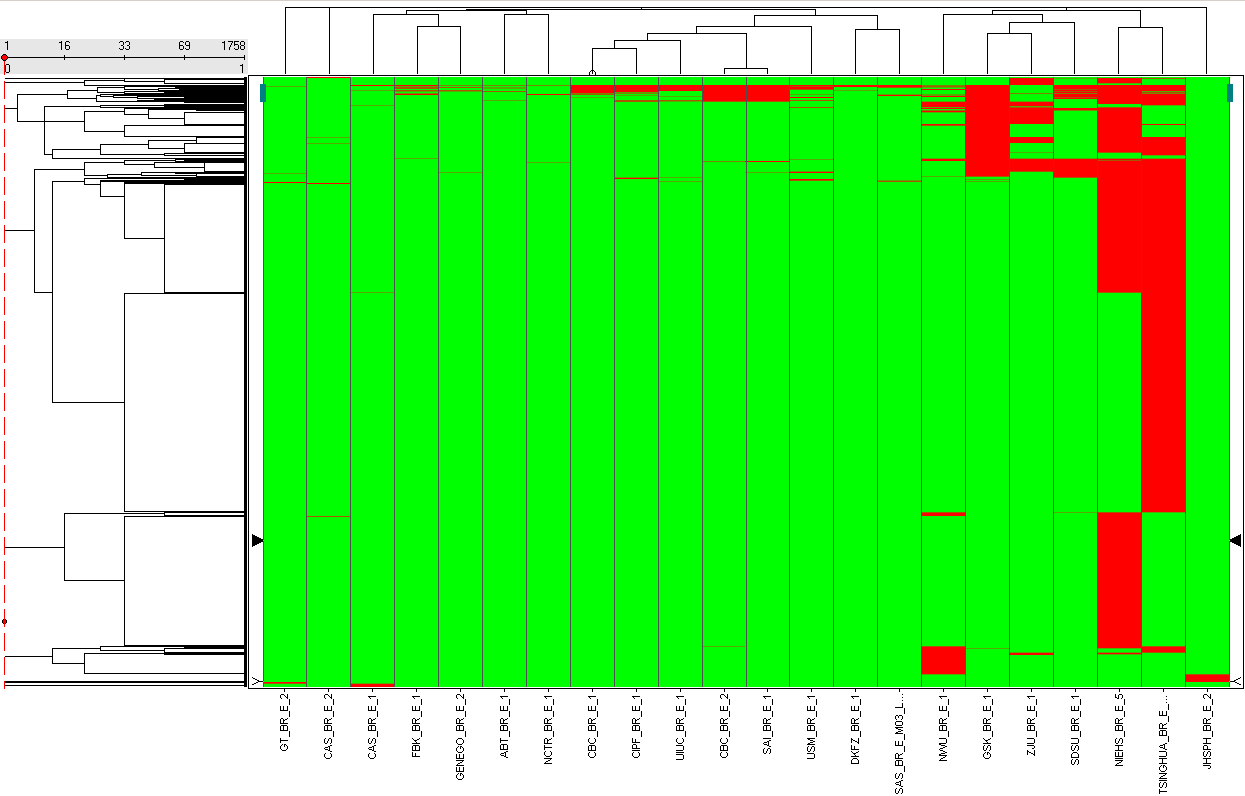

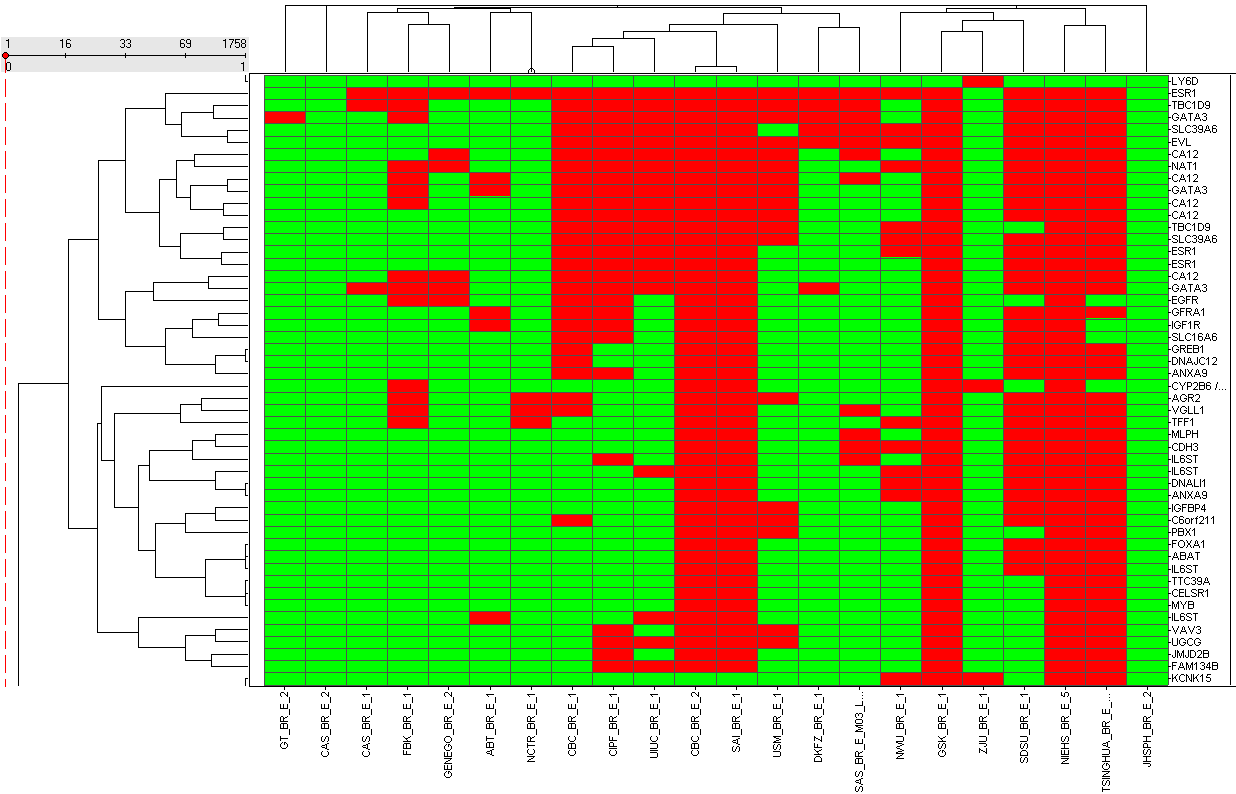


These two figures are heatmaps for endpoint E. The left one is the heatmap for all of the probes, and the right one is highlighted in top heatmap (Red => Probesets found in one or more models for endpoint E, Green => not).
