# Supplementary material for: Maximizing biomarker discovery by minimizing gene signatures
Source: BMC Genomics. 2011 Dec 23;12(Suppl 5):S6. doi: 10.1186/1471-2164-12-S5-S6 (PMC3287502; doi:10.1186/1471-2164-12-S5-S6)
Supplement: Additional file 6 — Internal and external validation for endpoint E. [file 1471-2164-12-S5-S6-S6.doc]

**Figure S2: Internal and external validation for endpoint E.**


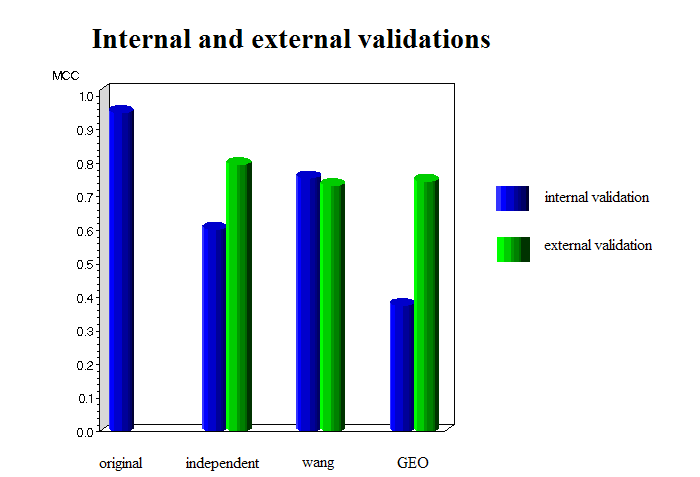


the blue column here refers to internal validation for each dataset. We utilized training set to predict the other dataset as external validations (the green column). Here classification algorithms for all validations are SVM. AGC method was proposed here for all datasets.
