# Supplementary material for: Maximizing biomarker discovery by minimizing gene signatures
Source: BMC Genomics. 2011 Dec 23;12(Suppl 5):S6. doi: 10.1186/1471-2164-12-S5-S6 (PMC3287502; doi:10.1186/1471-2164-12-S5-S6)

**Figure S5: Heatmaps for original models and swap models on training dataset and validation dataset**

Parameters for each sub-figure is followed: (a) BR_D_Model, training dataset; (b) BR_D_Model, validation dataset; (c) Swap_BR_D_Model, training dataset; (d) Swap_BR_D_Model, validation dataset; (e) BR_E_Model, training dataset; (f) BR_E_Model, validation dataset; (g) Swap_BR_E_Model, training dataset; and (h) Swap_BR_E_Model, validation dataset. Each column represents a sample in the dataset, and each row represents a gene in the gene signature. Note that the end row is endpoint info, i.e. pCR or erpos for endpoint D and endpoint E, respectively. Besides, if names for multiple genes that corresponds to a probe are too long, only one would listed.

Sub-figures for endpoint D, that is, (a), (b), (c) and (d), could not explicitly classified the samples into two groups, as for the reason that the distribution for sample was biased; However, samples who have evidence of residual disease (RD) can be aggregated. For endpoint E, the selected signatures can works well.


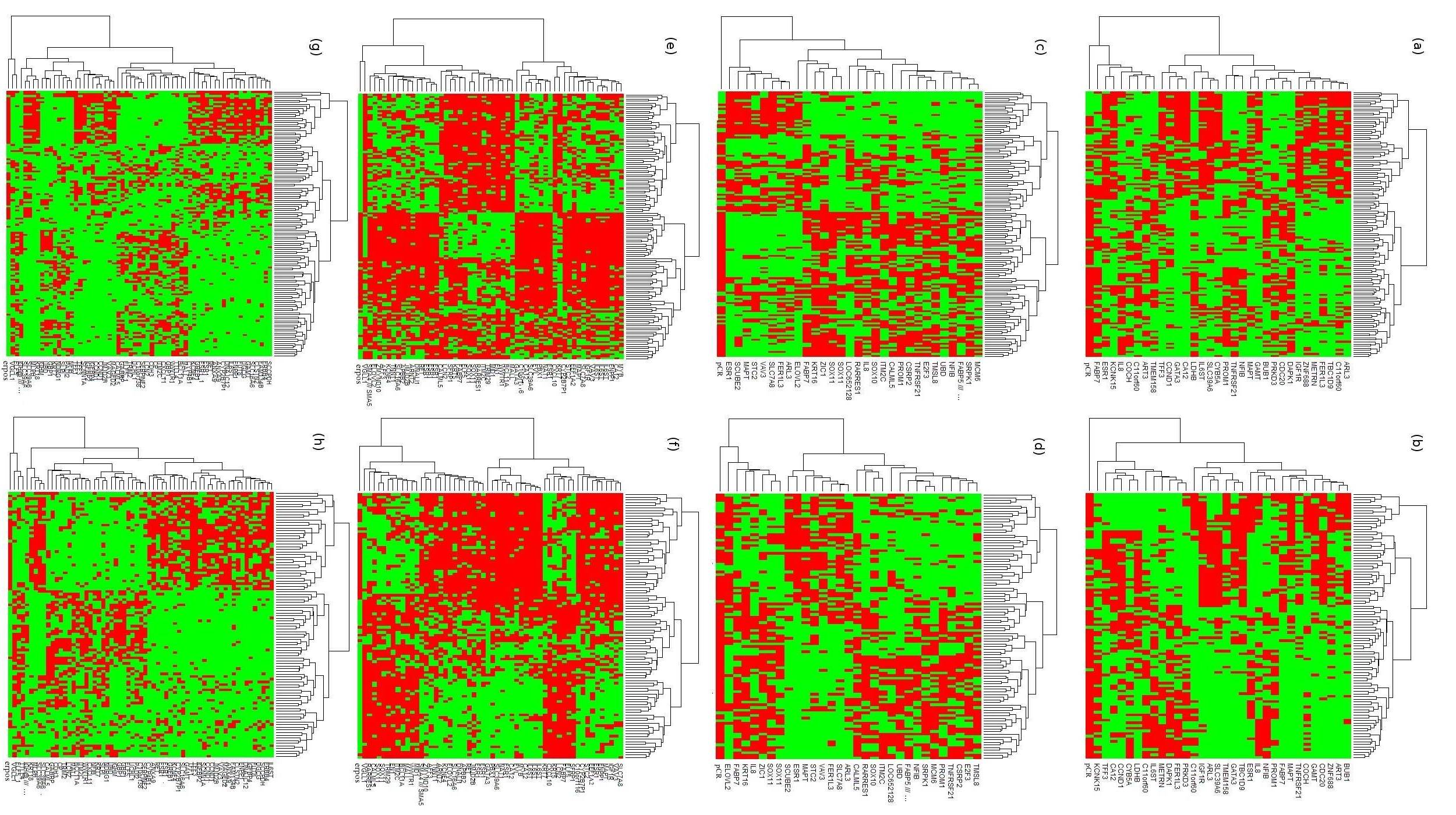

Supplement: Additional file 7 — Heatmaps for original models and swap models on training dataset and validation dataset. [file 1471-2164-12-S5-S6-S7.doc]
