# Supplementary material for: Maximizing biomarker discovery by minimizing gene signatures
Source: BMC Genomics. 2011 Dec 23;12(Suppl 5):S6. doi: 10.1186/1471-2164-12-S5-S6 (PMC3287502; doi:10.1186/1471-2164-12-S5-S6)
Supplement: Additional file 8 — Top genes (Similarity Analysis). [file 1471-2164-12-S5-S6-S8.doc]

**Table S4: Top genes (Similarity Analysis)**

| Endpoint D | | Endpoint E | | Endpoint D | | Endpoint E | | Endpoint D | | Endpoint E | |
| --- | --- | --- | --- | --- | --- | --- | --- | --- | --- | --- | --- |
| Gene | Time points | Gene | Time points | Gene | Time points | Gene | Time points | Gene | Time points | Gene | Time points |
| CA12 | 67 | CA12 | 61 | CXCR4 | 11 | VAV3 | 12 | ANP32E | 7 | IGFBP4 | 8 |
| MAPT | 43 | ESR1 | 53 | GREB1 | 11 | BCL11A | 11 | CD24 | 7 | SFRP1 | 8 |
| ESR1 | 33 | GATA3 | 40 | SCUBE2 | 11 | VGLL1 | 11 | COCH | 7 | STC2 | 8 |
| GATA3 | 25 | IL6ST | 33 | BCL11A | 10 | AGR2 | 10 | DAPK1 | 7 | TFF3 | 8 |
| BTG3 | 22 | SLC39A6 | 25 | CYP2B7P1 | 10 | CYP2B7P1 | 10 | EN1 | 7 | APBB2 | 7 |
| NFIB | 22 | TBC1D9 | 25 | SLC7A8 | 10 | DACH1 | 10 | FOXA1 | 7 | BTF3 | 7 |
| LDHB | 21 | MAPT | 22 | VGLL1 | 10 | ELF5 | 10 | GTSE1 | 7 | CX3CL1 | 7 |
| RARRES1 | 20 | NFIB | 20 | ART3 | 9 | GFRA1 | 10 | JMJD2B | 7 | DNALI1 | 7 |
| CCND1 | 18 | RARRES1 | 19 | CYB5A | 9 | MED13L | 10 | MLPH | 7 | GBP1 | 7 |
| ELF5 | 16 | ABAT | 16 | MCM5 | 9 | PBX1 | 10 | NAT1 | 7 | GREB1 | 7 |
| IGF1R | 16 | ANXA9 | 15 | METRN | 9 | SPDEF | 10 | SLC43A3 | 7 | HDGFRP3 | 7 |
| AGR2 | 15 | JMJD2B | 15 | DNAJC12 | 8 | TFF1 | 10 | STC2 | 7 | MLPH | 7 |
| TBC1D9 | 15 | EVL | 14 | GAMT | 8 | TRIM29 | 10 | TM4SF1 | 7 | NTN2L | 7 |
| GFRA1 | 14 | IL8 | 14 | IL8 | 8 | C6orf211 | 9 | TMEM158 | 7 | RARA | 7 |
| IL6ST | 14 | NAT1 | 14 | PADI2 | 8 | CDH3 | 9 | TNFRSF21 | 7 | SCCPDH | 7 |
| SOX11 | 14 | SOX11 | 14 | PTGER3 | 8 | EGFR | 9 | ARL3 | 6 | SLC16A6 | 7 |
| VAV3 | 14 | IGF1R | 13 | SLC39A6 | 8 | PTP4A2 | 9 | E2F3 | 6 | WWTR1 | 7 |
| IGFBP4 | 13 | FAM134B | 12 | SOX10 | 8 | TOX3 | 9 | FER1L3 | 6 | BTG3 | 6 |
| MELK | 12 | TRIM2 | 12 | TMSL8 | 8 | CSRP2 | 8 | ID4 | 6 | BUB3 | 6 |
| ABAT | 11 | UGCG | 12 | TTK | 8 | DNAJC12 | 8 | PROM1 | 6 | C10orf116 | 6 |
| Endpoint D | | Endpoint E | | Endpoint D | | Endpoint E | |  | | | |
| Gene | Time points | Gene | Time points | Gene | Time points | Gene | Time points |  | | | |
| SERPINE2 | 6 | C10orf38 | 6 | TIMP3 | 5 | VEGFA | 6 |  | | | |
| SFRP1 | 6 | CALML5 | 6 | TRIM2 | 5 | VEZF1 | 6 |  | | | |
| TFF3 | 6 | CCND1 | 6 | UGT8 | 5 | ZIC1 | 6 |  | | | |
| BUB1 | 5 | CD24 | 6 | ZNF688 | 5 | ABHD2 | 5 |  | | | |
| C11orf60 | 5 | CSNK1A1 | 6 | AMFR | 4 | AFF3 | 5 |  | | | |
| CENPA | 5 | DSC2 | 6 | C10orf116 | 4 | ASS1 | 5 |  | | | |
| CHI3L2 | 5 | ELOVL2 | 6 | CDC20 | 4 | C1orf106 | 5 |  | | | |
| CSRP2 | 5 | ENPP1 | 6 | CDH3 | 4 | C1orf34 | 5 |  | | | |
| ELOVL2 | 5 | ERBB4 | 6 | CTBP2 | 4 | CDKN2A | 5 |  | | | |
| GABRP | 5 | FOXA1 | 6 | CXCL11 | 4 | CELSR1 | 5 |  | | | |
| GPM6B | 5 | GPM6B | 6 | CXCL14 | 4 | CXCL10 | 5 |  | | | |
| KCNK15 | 5 | ID4 | 6 | CYP4B1 | 4 | CXCL11 | 5 |  | | | |
| MYO10 | 5 | LAMP3 | 6 | DSC2 | 4 | CYB5A | 5 |  | | | |
| PLAGL1 | 5 | LDHB | 6 | EPHB3 | 4 | EN1 | 5 |  | | | |
| RAB31 | 5 | PCM1 | 6 | FABP7 | 4 | FABP7 | 5 |  | | | |
| SEMA3C | 5 | PROM1 | 6 | FOLH1 | 4 | GABRE | 5 |  | | | |
| SKP1 | 5 | RET | 6 | HOMER3 | 4 | GABRP | 5 |  | | | |
| SKP2 | 5 | SCUBE2 | 6 | HRASLS | 4 | GAMT | 5 |  | | | |
| SSH3 | 5 | SEMA3F | 6 | IL12RB2 | 4 | IGHM | 5 |  | | | |
| TFF1 | 5 | TNFRSF21 | 6 | KIF2C | 4 | IGL@ | 5 |  | | | |
