# Supplementary material for: Maximizing biomarker discovery by minimizing gene signatures
Source: BMC Genomics. 2011 Dec 23;12(Suppl 5):S6. doi: 10.1186/1471-2164-12-S5-S6 (PMC3287502; doi:10.1186/1471-2164-12-S5-S6)
Supplement: Additional file 9 — GO term enrichment for overlapping genes (Similarity Analysis). [file 1471-2164-12-S5-S6-S9.doc]

**Table S5. GO term enrichment for overlapping genes (Similarity Analysis)**

| Endpoint | D | | | E | | |
| --- | --- | --- | --- | --- | --- | --- |
| GO  Ontology | biological  process | cellular  component | molecular  function | biological  process | cellular  component | molecular  function |
| GO id | 0031323 | 0044424 | 0043565 | 0031323 | 0044424 | 0003779 |
| Go term name | regulation of  cellular metabolic  process | intracellular  part | sequence-  specific DNA  binding | regulation of  cellular metabolic  process | intracellular  part | actin  binding |
| Background genes | 2021 | 6846 | 463 | 2021 | 6846 | 251 |
| Changed genes | 87 | 260 | 26 | 122 | 364 | 20 |
| p-value | 0.0158 | 0.0074 | 0.0108 | 0.0103 | 0.0176 | 0.0259 |
| enrichment | 1.24 | 1.09 | 1.62 | 1.21 | 1.07 | 1.6 |
| FDR | 0.1964 | 0.1374 | 0.154 | 0.3276 | 0.3479 | 0.4265 |

GO enrichment analysis (5^th^ level) for endpoints D and E.
