# Supplementary material for: Maximizing biomarker discovery by minimizing gene signatures
Source: BMC Genomics. 2011 Dec 23;12(Suppl 5):S6. doi: 10.1186/1471-2164-12-S5-S6 (PMC3287502; doi:10.1186/1471-2164-12-S5-S6)
Supplement: Additional file 10 — All pairs shortest path matrix counting for features before and after MFS. [file 1471-2164-12-S5-S6-S10.doc]

**Table S6: All pairs shortest path matrix counting for features before** and after MFS

| ModelID | CAS_BR_D_4 | | | | CAS_BR_E_15 | | | |
| --- | --- | --- | --- | --- | --- | --- | --- | --- |
| Item | Before MFS | | After MFS | | Before MFS | | After MFS | |
|  | Distance | Numbers | Distance | Numbers | Distance | Numbers | Distance | Numbers |
| Value | absent | 3675 | absent | 376 | absent | 18424 | absent | 1426 |
|  | no-link | 679 | no-link | 54 | no-link | 3751 | no-link | 287 |
|  | 1 | 8 | 1 | 1 | 1 | 19 | 1 | 3 |
|  | 2 | 77 | 2 | 8 | 2 | 237 | 2 | 21 |
|  | 3 | 259 | 3 | 24 | 3 | 1202 | 3 | 119 |
|  | 4 | 208 | 4 | 22 | 4 | 1016 | 4 | 99 |
|  | 5 | 43 | 5 | 10 | 5 | 197 | 5 | 25 |
|  | 6 | 1 | 6 | 1 | 6 | 4 | 6 | 1 |
| Average Distance | 3.342 | | 3.530 | | 3.413 | | 3.479 | |

In Distance column, the numbers refer to the distance between each two probes in a list of features. ‘absent’ means that one or two of the probe pair are absent. ‘no-link’ means that the pair is not connected in PPI network. Average distance for each item was calculated by using distance as weight.
