# Supplementary material for: Maximizing biomarker discovery by minimizing gene signatures
Source: BMC Genomics. 2011 Dec 23;12(Suppl 5):S6. doi: 10.1186/1471-2164-12-S5-S6 (PMC3287502; doi:10.1186/1471-2164-12-S5-S6)
Supplement: Additional file 11 — Datasets. [file 1471-2164-12-S5-S6-S11.doc]

Table S7: Datasets

| abbr | Resource | Acc.No. | Platform | Endpoint | Samples | Positive | Negative | P/N |
| --- | --- | --- | --- | --- | --- | --- | --- | --- |
| training | MAQC-II [1] |  | U133A | pCR | 130 | 33 | 97 | 0.34 |
|  |  |  |  | erpos | 130 | 80 | 50 | 1.6 |
| validation | MAQC-II [1] |  | U133A | pCR | 100 | 15 | 85 | 0.18 |
|  |  |  |  | erpos | 100 | 61 | 39 | 1.56 |
|  | Moggs JG et al [2] | GDS1326 | U133A | erpos | 12 | 0 | 12 | 0 |
|  | Farmer P et al [3] | GDS1329 | U133A | erpos | 49 | 27 | 22 | 1.23 |
|  | Lin CY et al [4] | GDS3217 | U133A, U133B | erpos | 18 | 18 | 0 | NaN |
| EV1 |  |  |  | erpos | 79 | 45 | 34 | 1.32 |
| EV2 | Wang et al [5] | GSE2034 | U133A | erpos | 286 | 209 | 77 | 2.71 |

EV1 dataset is the combination of the first three datasets since these three datasets were too small and the P/N ratios are too extreme.

1. Hess K, Anderson K, Symmans W, Valero V, Ibrahim N, Mejia J, Booser D, Theriault RL, Buzdar AU, Dempsey PJ *et al*: **Pharmacogenomic predictor of sensitivity to preoperative chemotherapy with paclitaxel and fluorouracil, doxorubicin, and cyclophosphamide in breast cancer**. *J Clin Oncol* 2006, **24**:4236-4244.

2. Moggs J, Murphy T, Lim F, Moore D, Stuckey R, Antrobus K, Kimber I, Orphanides G: **Anti-proliferative effect of estrogen in breast cancer cells that re-express ERalpha is mediated by aberrant regulation of cell cycle genes**. *J Mol Endocrinol* 2005, **34**:535-551.

3. Farmer P, Bonnefoi H, Becette V, Tubiana-Hulin M, Fumoleau P, Larsimont D, Macgrogan G, Bergh J, Cameron D, Goldstein D *et al*: **Identification of molecular apocrine breast tumours by microarray analysis**. *Oncogene* 2005, **24**:4660-4671.

4. Lin C, Vega V, Thomsen J, Zhang T, Kong S, Xie M, Chiu K, Lipovich L, Barnett DH, Stossi F *et al*: **Whole-genome cartography of estrogen receptor alpha binding sites**. *PLoS Genet* 2007, **3**:e87.

5. Wang Y, Klijn JGM, Zhang Y, Sieuwerts AM, Look MP, Yang F, Talantov D, Timmermans M: **Gene-expression profiles to predict distant metastasis of lymph-node-negative primary breast cancer**. *Lancet* 2005, **365**:671-679
