# Supplementary material for: Maximizing biomarker discovery by minimizing gene signatures
Source: BMC Genomics. 2011 Dec 23;12(Suppl 5):S6. doi: 10.1186/1471-2164-12-S5-S6 (PMC3287502; doi:10.1186/1471-2164-12-S5-S6)
Supplement: Additional file 12 — All pCR models as the input of CAS_BR_D_4. [file 1471-2164-12-S5-S6-S12.doc]

**Table S8** : All pCR models as the input of CAS_BR_D_4

| Unique Model  ID | Best Model | MCC | Accuracy | Sensitivity | Specificity | AUC | RMSE | MCC_Std Dev | Accuracy_Std Dev | Sensitivity_Std Dev | Specificity_Std Dev | AUC_Std Dev | RMSE_Std Dev | Summary Normalization | Feature Selection Method | Number of Features Used | Classification Algorithm | Batch Effect Removal Method | InternalValidation | ValidationIterations | Val_MCC | Val_Accuracy | Val_Sensitivity | Val_Specificity | Val_RMSE |
| --- | --- | --- | --- | --- | --- | --- | --- | --- | --- | --- | --- | --- | --- | --- | --- | --- | --- | --- | --- | --- | --- | --- | --- | --- | --- |
| ABT_BR_D_1 | Y | 0.458 | 0.7585 | 0.7424 | 0.7639 | 0.7532 | 0.4911 | 0.0482 | 0.0182 | 0.0558 | 0.0178 | 0.0282 | 0.0188 | MAS5 | SAM | 6 | DA | None | 5-CV | 10 | 0.3331 | 0.68 | 0.8 | 0.6588 | 0.5657 |
| CAS_BR_D_1 | Y | 0.8677 | 0.9391 | 0.9505 | 0.9386 | 0.9646 |  | 0.0607 | 0.0276 | 0.0551 | 0.0417 | 0.0311 |  | MAS5 | RFE | 11 | SVM | None | 5-CV | 10 | 0.3361 | 0.75 | 0.6667 | 0.7647 | 0.5 |
| CAS_BR_D_2 | N | 0.7011 | 0.8681 | 0.9284 | 0.85 |  |  | 0.1217 | 0.0603 | 0.0862 | 0.0685 |  |  | MAS5 | Pathway | 10 | NB | None | 5-CV | 10 | 0.3122 | 0.73 | 0.6667 | 0.7412 | 0.5196 |
| CBC_BR_D_1 | Y | 0.536 | 0.825 | 0.557 | 0.923 |  |  | 0.0499 | 0.0164 | 0.0319 | 0.0156 |  |  | MAS5 | SAM | 22 | SVM | None | 5-CV | 10 | 0.324 | 0.74 | 0.6667 | 0.7529 | 0.5036 |
| CBC_BR_D_2 | N | 0.4149 | 0.7692 | 0.6333 | 0.81 |  |  | 0.1422 | 0.0608 | 0.1394 | 0.0652 |  |  | MAS5 | SAM | 38 | KNN | None | 5-CV | 10 | 0.2634 | 0.72 | 0.6 | 0.7412 | 0.5112 |
| CDRH_BR_D_4 | Y | 0.344 | 0.772 | 0.455 | 0.871 | 0.796 | 0.418 | 0.188 | 0.064 | 0.185 | 0.064 | 0.084 | 0.064 | refRMA | eGOMiner | 25 | Logistic | None | 5-CV | 10 | 0.2605 | 0.6 | 0.8 | 0.5647 | 0.5785 |
| CIPF_BR_D_1 | Y | 0.5267 | 0.7969 | 0.7636 | 0.8082 | 0.7859 |  | 0.0311 | 0.0137 | 0.0239 | 0.0121 | 0.0164 |  | MAS5 | Wilcoxon | 90 | DA | None | 5-CV | 10 | -0.2482 | 0.37 | 0.2667 | 0.3882 | 0.7937 |
| DKFZ_BR_D_1 | Y | 0.213 | 0.7296 | 0.31 | 0.877 | 0.681 |  | 0.108 | 0.0281 | 0.129 | 0.043 | 0.042 |  | VSN+RMA | PAM | 13 | PAM | None | 5-CV | 10 | 0.3177 | 0.79 | 0.5333 | 0.8353 | 0.4583 |
| FBK_BR_D_1 | N | 0.409 | 0.78 | 0.535 | 0.863 | 0.848 |  | 0.178 | 0.059 | 0.187 | 0.073 | 0.061 |  | MAS5 | RFE | 10 | DA | None | 5-CV | 10 | 0.3635 | 0.71 | 0.8 | 0.6941 | 0.5385 |
| FBK_BR_D_2 | N | 0.328 | 0.746 | 0.476 | 0.84 | 0.749 |  | 0.188 | 0.073 | 0.198 | 0.085 | 0.103 |  | MAS5 | RFE | 100 | SVM | None | 5-CV | 10 | 0.2102 | 0.74 | 0.4667 | 0.7882 | 0.5099 |
| GeneGo_BR_D_3 | Y | 0.4734 | 0.7568 | 0.7733 | 0.7522 | 0.8168 | 0.4311 | 0.0557 | 0.0297 | 0.0443 | 0.0365 | 0.0327 | 0.0212 | RMA | FC+P | 9 | DA | P.Rank | 5-CV | 10 | 0.3047 | 0.65 | 0.8 | 0.6235 | 0.4966 |
| GSK_BR_D_1 | Y | 0.427 | 0.748 | 0.71 | 0.761 | 0.828 |  | 0.028 | 0.014 | 0.027 | 0.017 | 0.002 |  | MAS5 | FC+P | 3 | NB | Mean Shift | 5-CV | 10 | 0.3058 | 0.69 | 0.7333 | 0.6824 | 0.4913 |
| GT_BR_D_4 | Y | 0.3006 | 0.7476 | 0.4243 | 0.8566 | 0.7359 | 0.4322 | 0.2334 | 0.0836 | 0.2245 | 0.0901 | 0.1291 | 0.075 | PLIER+caCORRECT | GeneticAlgorithm | 10 | SVM | None | 5-CV | 10 | 0.1896 | 0.81 | 0.2667 | 0.9059 | 0.3704 |
| JHSPH_BR_D_2 | Y | 0.46 | 0.78 | 0.68 | 0.81 | 0.81 | 0.47 | 0.043 | 0.016 | 0.047 | 0.015 | 0.013 | 0.017 | refRMA | Barcode | 24 | Barcode | Barcode | 5-CV | 50 | 0.2667 | 0.65 | 0.7333 | 0.6353 | 0.4631 |
| NCTR_BR_D_1 | Y | 0.3651 | 0.7481 | 0.8133 | 0.5619 | 0.7398 |  | 0.0372 | 0.0167 | 0.0189 | 0.0301 | 0.0336 |  | MAS5 | FC+P | 5 | NB | Mean Shift | 5-CV | 10 | 0.2287 | 0.65 | 0.6667 | 0.6471 | 0.5916 |
| NIEHS_BR_D_8 | Y | 0.2687 | 0.7622 | 0.2543 | 0.94 |  | 0.2423 | 0.0817 | 0.043 | 0.169 | 0.0505 |  | 0.0274 | SVN | FC+P | 68 | SVM | ComBat | 5-CV | 10 | 0.3238 | 0.86 | 0.2667 | 0.9647 | 0.3742 |
| NWU_BR_D_1 | Y | 0.3122 | 0.7625 | 0.353 | 0.9075 | 0.8073 | 0.3897 | 0.1785 | 0.0665 | 0.1882 | 0.0622 | 0.0896 | 0.0431 | MAS5 | FC+P | 1622 | Tree | None | 5-CV | 10 | 0.2885 | 0.77 | 0.5333 | 0.8118 | 0.3827 |
| NWU_BR_D_13 | N | 0.2836 | 0.7513 | 0.3652 | 0.8811 | 0.754 | 0.4206 | 0.1943 | 0.0825 | 0.1968 | 0.0759 | 0.0953 | 0.0644 | MAS5 | FC+P | 15 | SVM | None | 5-CV | 10 | 0.3863 | 0.83 | 0.5333 | 0.8824 | 0.3671 |
| Roche_BR_D_1 | Y | 0.442 | 0.8061 | 0.48 | 0.9154 | 0.6977 |  | 0.0635 | 0.0174 | 0.0183 | 0.0276 | 0.0069 |  | MAS5 | SA | 10 | Tree | None | 10-CV | 10 | 0.2749 | 0.76 | 0.5333 | 0.8 | 0.4899 |
| SAI_BR_D_1 | Y | 0.51 | 0.781 | 0.76 | 0.79 |  |  | 0.16 | 0.071 | 0.152 | 0.082 |  |  | RMA | Welch | 68 | ML | None | 5-CV | 40 | 0.3234 | 0.67 | 0.8 | 0.6471 | 0.5702 |
| SAS_BR_D_M23_PLS_023 | Y | 0.3814 | 0.7815 | 0.4545 | 0.8928 | 0.832 | 0.3793 | 0.021 | 0.0074 | 0.0202 | 0.0087 | 0.0077 | 0.0032 | Mean | T-Test | 120 | PLS | None | 5-CV | 10 | 0.3603 | 0.74 | 0.7333 | 0.7412 | 0.5099 |
| SDSU_BR_D_1 | Y | 0.5468 | 0.6531 | 0.7577 | 0.8312 |  |  | 0.2136 | 0.0841 | 0.2174 | 0.0983 |  |  | MAS5+Loess | FC+P | 30 | KNN | Mean Shift | 5-CV | 10 | 0.3395 | 0.78 | 0.6 | 0.8118 | 0.4273 |
| SDSU_BR_D_2 | N | 0.5232 | 0.7378 | 0.7195 | 0.8302 |  |  | 0.196 | 0.0762 | 0.1985 | 0.0783 |  |  | MAS5+Loess | FC+P | 50 | PM | Mean Shift | 5-CV | 10 | 0.3058 | 0.69 | 0.7333 | 0.6824 | 0.499 |
| Spheromics_BR_D_1 | Y | 0.4327 | 0.7747 | 0.569 | 0.8467 | 0.8629 | 0.3663 | 0.0434 | 0.0174 | 0.0362 | 0.0159 | 0.0096 | 0.0058 | MAS5 | Permutation | 206 | PLS | None | 5-CV | 20 | 0.3008 | 0.72 | 0.6667 | 0.7294 | 0.4086 |
| Tsinghua_BR_D_6 | Y | 0.3672 | 0.7708 | 0.4788 | 0.8701 | 0.7541 | 0.4055 | 0.0734 | 0.0237 | 0.0818 | 0.0229 | 0.0295 | 0.0138 | dChip | FC+P | 44 | KNN | None | 5-CV | 10 | -0.0422 | 0.84 | 0 | 0.9882 | 0.3493 |
| UIUC_BR_D_1 | Y | 0.6661 | 0.8623 | 0.8333 | 0.8722 | 0.8527 | 0.3664 | 0.0579 | 0.0242 | 0.0434 | 0.0434 | 0.0296 | 0.0319 | MAS5 | T-Test | 18 | KNN | None | 5-CV | 10 | 0.2029 | 0.7 | 0.5333 | 0.7294 | 0.5436 |
| USM_BR_D_1 | Y | 0.5733 | 0.8177 | 0.8342 | 0.7719 | 0.8675 | 0.4149 | 0.0238 | 0.0081 | 0.0104 | 0.02 | 0.0093 | 0.0086 | MAS5 | FC+P | 50 | NB | None | 5-CV | 10 | 0.3488 | 0.73 | 0.7333 | 0.7294 | 0.5196 |
| ZJU_BR_D_1 | Y | 0.4437 | 0.8054 | 0.4758 | 0.9175 |  |  | 0.0167 | 0.0063 | 0.0205 | 0.0109 |  |  | MAS5 | FC+P | 40 | Nearest Centroid | None | 5-CV | 10 | 0.3851 | 0.73 | 0.8 | 0.7176 | 0.5196 |

**Red Numbers are corrected to its right number.**
