# Supplementary material for: Maximizing biomarker discovery by minimizing gene signatures
Source: BMC Genomics. 2011 Dec 23;12(Suppl 5):S6. doi: 10.1186/1471-2164-12-S5-S6 (PMC3287502; doi:10.1186/1471-2164-12-S5-S6)
Supplement: Additional file 13 — All ER models as the input of CAS_BR_E_15. [file 1471-2164-12-S5-S6-S13.doc]

**Table S9: All ER** models as the input of CAS_BR_E_15

| UniqueModel  ID | Model Rank Order | Best Model | MCC | Accuracy | Sensitivity | Specificity | AUC | RMSE | MCC_StdDev | Accuracy_StdDev | Sensitivity_StdDev | Specificity_StdDev | AUC_StdDev | RMSE_StdDev | Summary Normalization | Feature Selection Method | Number of Features Used | ClassificationAlgorithm | Batch Effect Removal Method | Internal Validation | Validation Iterations | Val_MCC | Val_Accuracy | Val_Sensitivity | Val_Specificity | Val_AUC | Val_RMSE |
| --- | --- | --- | --- | --- | --- | --- | --- | --- | --- | --- | --- | --- | --- | --- | --- | --- | --- | --- | --- | --- | --- | --- | --- | --- | --- | --- | --- |
| GSK_BR_E_1 | 1 | Y | 0.848 | 0.928 | 0.938 | 0.912 | 0.973 |  | 0.014 | 0.007 | 0.009 | 0.011 | 0.003 |  | MAS5 | FC+P | 316 | NB | Mean Shift | 5-CV | 10 | 0.7924 | 0.9 | 0.9016 | 0.8974 |  | 0.3148 |
| GeneGo_BR_E_2 | 3 | Y | 0.8555 | 0.9316 | 0.9725 | 0.866 | 0.9631 | 0.2503 | 0.0096 | 0.0042 | 0.01 | 0.0135 | 0.0053 | 0.003 | RMA | FC+P | 7 | DA | P.Rank | 5-CV | 10 | 0.7782 | 0.89 | 0.8689 | 0.9231 |  | 0.3086 |
| NWU_BR_E_1 | 1 | Y | 0.8669 | 0.94 | 0.9786 | 0.8704 | 0.9787 | 0.2467 | 0.1006 | 0.0427 | 0.0348 | 0.116 | 0.034 | 0.0474 | MAS5 | FC+P | 141 | Tree | None | 5-CV | 10 | 0.7735 | 0.89 | 0.8852 | 0.8974 |  | 0.3186 |
| ZJU_BR_E_1 | 1 | Y | 0.8743 | 0.94 | 0.985 | 0.88 |  |  | 0.0207 | 0.0101 | 0.0053 | 0.027 |  |  | MAS5 | FC+P | 145 | KNN | None | 5-CV | 10 | 0.7735 | 0.89 | 0.8852 | 0.8974 |  | 0.3317 |
| FBK_BR_E_1 | 2 | N | 0.89 | 0.947 | 0.966 | 0.916 | 0.981 |  | 0.065 | 0.031 | 0.038 | 0.064 | 0.026 |  | MAS5 | RFE | 15 | SVM | None | 5-CV | 10 | 0.7735 | 0.89 | 0.8852 | 0.8974 |  | 0.3317 |
| USM_BR_E_1 | 1 | Y | 0.9211 | 0.9615 | 0.9825 | 0.928 | 0.9553 | 0.1531 | 0.013 | 0.0063 | 0.0065 | 0.014 | 0.0073 | 0.0277 | MAS5 | FC+P | 30 | SMO | None | 5-CV | 10 | 0.77 | 0.89 | 0.9016 | 0.8718 |  | 0.3317 |
| Tsinghua_BR_E_50 | 2 | Y | 0.8733 | 0.94 | 0.975 | 0.884 | 0.9671 | 0.2337 | 0.0212 | 0.0101 | 0.0059 | 0.0295 | 0.005 | 0.0072 | dChip | SAM | 1150 | KNN | None | 5-CV | 10 | 0.7549 | 0.88 | 0.8689 | 0.8974 |  | 0.3305 |
| NIEHS_BR_E_5 | 1 | Y | 0.9014 | 0.9531 | 0.9763 | 0.916 |  | 0.9214 | 0.0346 | 0.0375 | 0.0417 | 0.0842 |  | 0.0156 | SVN | FC+P | 984 | SVM | ComBat | 5-CV | 10 | 0.7478 | 0.88 | 0.9016 | 0.8462 |  | 0.3464 |
| SAI_BR_E_1 | 1 | Y | 0.905 | 0.954 | 0.918 | 0.977 |  |  | 0.075 | 0.036 | 0.083 | 0.035 |  |  | MAS5 | SAM | 51 | SVM | None | 5-CV | 40 | 0.7478 | 0.88 | 0.9016 | 0.8462 |  | 0.3377 |
| CAS_BR_E_2 | 2 | N | 0.8182 | 0.9154 | 0.9181 | 0.9237 | 0.9441 |  | 0.0569 | 0.0268 | 0.083 | 0.0426 | 0.0496 |  | MAS5 | RFE | 10 | SVM | None | 5-CV | 10 | 0.7429 | 0.87 | 0.8361 | 0.9231 |  | 0.3606 |
| CBC_BR_E_1 | 1 | Y | 0.913 | 0.958 | 0.984 | 0.916 |  |  | 0.0189 | 0.0093 | 0.008 | 0.0174 |  |  | MAS5 | SAM | 27 | SVM | None | 5-CV | 10 | 0.7368 | 0.87 | 0.8525 | 0.8974 |  | 0.3471 |
| CBC_BR_E_2 | 2 | N | 0.9214 | 0.9615 | 0.9882 | 0.9218 |  |  | 0.0554 | 0.0272 | 0.0263 | 0.0832 |  |  | MAS5 | SAM | 50 | KNN | None | 5-CV | 10 | 0.7337 | 0.86 | 0.8033 | 0.9487 |  | 0.364 |
| ABT_BR_E_1 | 1 | Y | 0.8699 | 0.9385 | 0.9713 | 0.886 | 0.9286 | 0.2475 | 0.0191 | 0.0089 | 0.0119 | 0.0165 | 0.0094 | 0.018 | MAS5 | SAM | 6 | DA | None | 5-CV | 10 | 0.7319 | 0.87 | 0.8689 | 0.8718 |  | 0.3358 |
| SDSU_BR_E_1 | 1 | Y | 0.9262 | 0.874 | 0.9809 | 0.9342 |  |  | 0.0685 | 0.0509 | 0.0389 | 0.0848 |  |  | MAS5+Loess | FC+P | 100 | PM | Mean Shift | 5-CV | 10 | 0.7176 | 0.85 | 0.7869 | 0.9487 |  | 0.3516 |
| JHSPH_BR_E_2 | 1 | Y | 0.81 | 0.91 | 0.92 | 0.89 | 0.97 | 0.3 | 0.024 | 0.011 | 0.016 | 0.021 | 0.005 | 0.02 | refRMA | Barcode | 22 | Barcode | Barcode | 5-CV | 50 | 0.7018 | 0.85 | 0.8197 | 0.8974 |  | 0.3575 |
| UIUC_BR_E_1 | 1 | Y | 0.9056 | 0.9554 | 0.9688 | 0.934 | 0.9799 | 0.2054 | 0.0103 | 0.0049 | 0.0066 | 0.0066 | 0.0008 | 0.0078 | MAS5 | T-Test | 23 | NB | None | 5-CV | 10 | 0.7018 | 0.85 | 0.8197 | 0.8974 |  | 0.3869 |
| GT_BR_E_2 | 1 | Y | 0.8079 | 0.9069 | 0.9338 | 0.864 | 0.9572 | 0.251 | 0.1244 | 0.0606 | 0.0666 | 0.1109 | 0.0399 | 0.0767 | MAS5 | GeneticAlgorithm | 10 | SVM | None | 5-CV | 10 | 0.6955 | 0.85 | 0.8361 | 0.8718 |  | 0.3572 |
| NCTR_BR_E_1 | 1 | Y | 0.8924 | 0.9469 | 0.9114 | 0.9739 | 0.9874 |  | 0.0233 | 0.0117 | 0.0284 | 0.0092 | 0.0033 |  | MAS5 | FC+P | 5 | NB | Mean Shift | 5-CV | 10 | 0.6904 | 0.85 | 0.8525 | 0.8462 |  | 0.3873 |
| SAS_BR_E_M03_LR_015 | 1 | Y | 0.8813 | 0.9438 | 0.9625 | 0.914 | 0.9733 | 0.2352 | 0.0189 | 0.0089 | 0.0144 | 0.0165 | 0.0016 | 0.0041 | Mean | T-Test | 14 | Logistic | None | 5-CV | 10 | 0.6517 | 0.82 | 0.7705 | 0.8974 |  | 0.4243 |
| CIPF_BR_E_1 | 1 | Y | 0.9024 | 0.9538 | 0.9675 | 0.932 | 0.9498 |  | 0.0232 | 0.0109 | 0.0105 | 0.0193 | 0.012 |  | MAS5 | Wilcoxon | 30 | PAM | None | 5-CV | 10 | 0.5734 | 0.77 | 0.6885 | 0.8974 |  | 0.4796 |
| CAS_BR_E_1 | 1 | Y | 0.8699 | 0.9385 | 0.9496 | 0.9231 |  |  | 0.1326 | 0.0607 | 0.0553 | 0.0972 |  |  | MAS5 | Pathway | 15 | NB | None | 5-CV | 10 | 0.5435 | 0.75 | 0.6557 | 0.8974 |  | 0.5 |
| DKFZ_BR_E_1 | 1 | Y | 0.871 | 0.9346 | 0.909 | 0.969 | 0.969 |  | 0.024 | 0.0117 | 0.017 | 0.025 | 0.019 |  | VSN+RMA | PAM | 6 | PAM | None | 5-CV | 10 | 0.4993 | 0.71 | 0.5738 | 0.9231 |  | 0.5385 |

**Red Numbers are corrected to its right number.**
