# Supplementary material for: Maximizing biomarker discovery by minimizing gene signatures
Source: BMC Genomics. 2011 Dec 23;12(Suppl 5):S6. doi: 10.1186/1471-2164-12-S5-S6 (PMC3287502; doi:10.1186/1471-2164-12-S5-S6)

**Document S1: Bayesian-Decision Tree Method**

The Bayesian Decision Tree transforms each probe to be classified to its closest gene by calculating posterior probability, using Bayesian Theory and posterior mean of probes related to the given probe.

We assumed a Dirichlet distribution, and posterior probabilities for each probe set and Pearson correlation coefficient (PCC) for all probes were calculated. If the PCC was greater than 0.9 and the p-value was less than 0.05, the gene was added to a new set, with the number of newly-generated sets counted (*n*). According to Bayesian theory, we calculated the posterior probability of a probe be classified to each gene. Finally, a Decision Tree with a series of thresholds was used to assign probes to most likely genes.

*p* was defined as:

$$p=\frac{\beta_{i}+X_{i}}{\sum\beta_{i}+\sum X_{i}}$$

$$X_{i}=betainv(n/N,n+a,N-n+b)\times N$$

Where *p* = the posterior mean of probability, *β_i_* = dirichlet distribution parameter, *betainv* = a function of inverse beta distribution; *n* = previously defined, *N* = the size of the probe set, and *a* and *b* are the parameters of the beta distribution. The following graph recorded the result of Bayesian-Decision Tree Method.


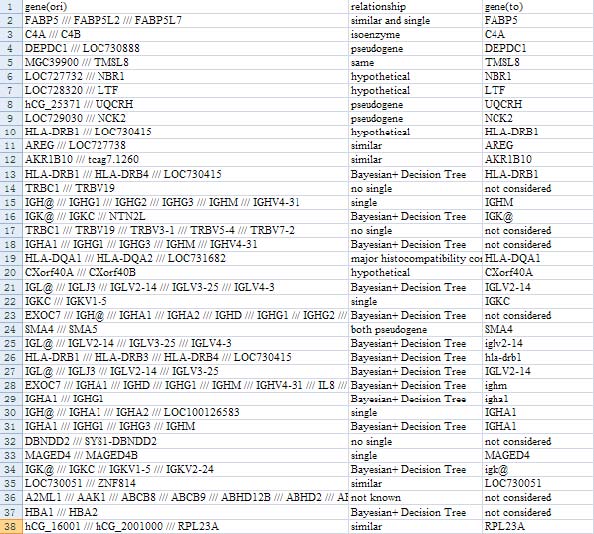

Supplement: Additional file 14 — Bayesian-Decision Tree Method. [file 1471-2164-12-S5-S6-S14.doc]
