# Supplementary material for: Maximizing biomarker discovery by minimizing gene signatures
Source: BMC Genomics. 2011 Dec 23;12(Suppl 5):S6. doi: 10.1186/1471-2164-12-S5-S6 (PMC3287502; doi:10.1186/1471-2164-12-S5-S6)
Supplement: Additional file 15 — Development of the Local Network Model (LNM) tool. [file 1471-2164-12-S5-S6-S15.doc]

**Document S3: Development of t**he **Local Network Model (LNM) tool**

We developed LNM, a tool for detecting overlapping probes at the gene expression level. Given that a large number of probe overlaps exist among the two breast cancer endpoints examined, detection of overlap in gene expression could be omitted.

Gene expression profile correlations analyses are based on probes and their expression values. Gene signature probes were treated as points in N-dimensional space based on their Euclidean distances based on gene expression profile, and each gene signature was treated as a sphere. First, a quality control process was executed. For each sphere, outliers were discarded. We then examined pairs of spheres, and those that were too close to each other were combined. Check process for outliers of the combined sphere would be restarted after the combination. All signatures examined in our study were placed in an N-dimensional space based on microarray expression profile values of features, with a point representing a single probe. In this N-dimensional space, if the difference between the distance between a point and the center of the space and the average distance from all points to the center is larger than twice the standard deviation, the point is considered an outlier.

Overlapping probes at expression level were calculated using the following rules. The center of a sphere was the geometric center for each point set. We first combined all points into a single set. For each point p_i_ in the union set, its distances to central O_1_ and O_2_ are d_i1_ and d_i2_, respectively. If r_1_+r_2_< O_1_O_2_ then no overlap was found. Otherwise, d_i1_<=r_1_ and d_i2_<=r_2_, and we would say that p_1_ is an overlapping probe. The radius was the maximal distance for a center of gravity to all points in a set. For a point p, if the distance between its Euclidean Distance and the average value for all Euclidean Distances in the gene signature is larger than 2 times of the standard deviations for all Euclidean distances, we call it an outlier. Two spheres are too close if O_1_O_2_< k (r_1_+r_2_), where we set k=0.05.
