# Supplementary material for: Maximizing biomarker discovery by minimizing gene signatures
Source: BMC Genomics. 2011 Dec 23;12(Suppl 5):S6. doi: 10.1186/1471-2164-12-S5-S6 (PMC3287502; doi:10.1186/1471-2164-12-S5-S6)
Supplement: Additional file 16 — MFS inputs. [file 1471-2164-12-S5-S6-S16.doc]

**Document S2: MFS inputs**

MFS can accomodate multiple inputs. For similarity analysis, we used overlapping probes as our input for MFS, as they are represented by various classifiers. However, it is also reasonable to use probes from other sources as input since the MFS process reduces redundancy, and performance is determined by the original gene signature.

Previous studies have suggested that little overlap exists among gene signatures for the same disease [1, 2]. We developed Local Network Model (LNM, see Methods), a method to identify overlapping probes at the gene expression profile level. This model would be especially useful for situations in which little or no overlap exists at the probe level. Overlapping probes were treated as input for MFS analysis, resulting in 0.553 (5-CV, training dataset) and 0.404 (validation dataset) for endpoint D and 0.951 (5-CV, training dataset) and 0.770 (validation dataset) for endpoint E, respectively. Furthermore, we also used another two gene signatures, CAS_BR_D_3 (Val_MCC=0.3122) and CAS_BR_E_9 (Val_MCC=0.5435) from MAQC-II submitted models for endpoints D and E individually as our input for MFS to validate the MFS method. The performance of the output classifiers were 0.816 (5-CV, training dataset) and 0.183 (validation dataset) for endpoint D and 0.935 (5-CV, training dataset) and 0.770 (validation dataset) for endpoint E, respectively.

1. Natsoulis G, Ghaoui LE, Lanckriet GRG, Tolley AM, Leroy F, Dunlea S, Eynon BP, Pearson CI, Tugendreich S, Jarnagin K: **Classification of a large microarray data set: Algorithm comparison and analysis of drug signatures**. *Genome Res* 2005, **15**:724-736.

2. Natsoulis G, Pearson C, Gollub J, P Eynon B, Ferng J, Nair R, Idury R, Lee M, Fielden M, Brennan R *et al*: **The liver pharmacological and xenobiotic gene response repertoire**. *Mol Syst Biol* 2008, **4**:175.
