# Supplementary material for: Maximizing biomarker discovery by minimizing gene signatures
Source: BMC Genomics. 2011 Dec 23;12(Suppl 5):S6. doi: 10.1186/1471-2164-12-S5-S6 (PMC3287502; doi:10.1186/1471-2164-12-S5-S6)
Supplement: Additional file 17 — Features of the CAS_BR_D_4 and CAS_BR_E_15. [file 1471-2164-12-S5-S6-S17.doc]

**Table S10: Features of the CAS_BR_D_4 and CAS_BR_E_15**

| CAS_BR_D_4 |  | CAS_BR_E_5 |  |  |
| --- | --- | --- | --- | --- |
| 213564_x_at | 209366_x_at | 205225_at | 202134_s_at | 215616_s_at |
| 203963_at | 211864_s_at | 214440_at | 220414_at | 202752_x_at |
| 209603_at | 203930_s_at | 214164_x_at | 204914_s_at | 215043_s_at |
| 212960_at | 204304_s_at | 217838_s_at | 213712_at | 201820_at |
| 210147_at | 218236_s_at | 209604_s_at | 203627_at | 202036_s_at |
| 204623_at | 211506_s_at | 202089_s_at | 222379_at | 204540_at |
| 203628_at | 213527_s_at | 210735_s_at | 204533_at | 205030_at |
| 217190_x_at | 213338_at | 215552_s_at | 209016_s_at | 212531_at |
| 209290_s_at | 205029_s_at | 211233_x_at | 211421_s_at | 202986_at |
| 205354_at |  | 211000_s_at | 220559_at |  |
| 219051_x_at |  | 215729_s_at | 220625_s_at |  |
| 203139_at |  | 204863_s_at | 203637_s_at |  |
| 208712_at |  | 203438_at | 205734_s_at |  |
| 220540_at |  | 203571_s_at | 211002_s_at |  |
| 218856_at |  | 203928_x_at | 213201_s_at |  |
| 205229_s_at |  | 206392_s_at | 205186_at |  |
| 212195_at |  | 206754_s_at | 204915_s_at |  |
| 202641_at |  | 209373_at | 211234_x_at |  |
| 218483_s_at |  | 218806_s_at | 213419_at |  |
| 202870_s_at |  | 212196_at | 219498_s_at |  |
| 209642_at |  | 212148_at | 221004_s_at |  |
| 201755_at |  | 205066_s_at | 205714_s_at |  |
| 202089_s_at |  | 207038_at | 204798_at |  |
